# Land use and climate change impacts on global soil erosion by water (2015-2070)

**DOI:** 10.1073/pnas.2001403117

**Published:** 2020-08-24

**Authors:** Pasquale Borrelli, David A. Robinson, Panos Panagos, Emanuele Lugato, Jae E. Yang, Christine Alewell, David Wuepper, Luca Montanarella, Cristiano Ballabio

**Affiliations:** ^a^Environmental Geosciences, University of Basel, Basel 4056, Switzerland;; ^b^Department of Biological Environment, Kangwon National University, Chuncheon 24341, Republic of Korea;; ^c^UK Centre for Ecology and Hydrology, Environment Centre Wales, Bangor LL57 2UW, United Kingdom;; ^d^European Commission, Joint Research Centre (JRC), Ispra (VR) 21027, Italy;; ^e^Agricultural Economics and Policy, ETH Zurich, Zurich 8092, Switzerland

**Keywords:** land degradation, agricultural sustainability, policy scenarios

## Abstract

Soil erosion is a major global soil degradation threat to land, freshwater, and oceans. Wind and water are the major drivers, with water erosion over land being the focus of this work; excluding gullying and river bank erosion. Improving knowledge of the probable future rates of soil erosion, accelerated by human activity, is important both for policy makers engaged in land use decision-making and for earth-system modelers seeking to reduce uncertainty on global predictions. Here we predict future rates of erosion by modeling change in potential global soil erosion by water using three alternative (2.6, 4.5, and 8.5) Shared Socioeconomic Pathway and Representative Concentration Pathway (SSP-RCP) scenarios. Global predictions rely on a high spatial resolution Revised Universal Soil Loss Equation (RUSLE)-based semiempirical modeling approach (GloSEM). The baseline model (2015) predicts global potential soil erosion rates of $43_{-7}^{+9.2}$ Pg yr^−1^, with current conservation agriculture (CA) practices estimated to reduce this by ∼5%. Our future scenarios suggest that socioeconomic developments impacting land use will either decrease (SSP1-RCP2.6–10%) or increase (SSP2-RCP4.5 +2%, SSP5-RCP8.5 +10%) water erosion by 2070. Climate projections, for all global dynamics scenarios, indicate a trend, moving toward a more vigorous hydrological cycle, which could increase global water erosion (+30 to +66%). Accepting some degrees of uncertainty, our findings provide insights into how possible future socioeconomic development will affect soil erosion by water using a globally consistent approach. This preliminary evidence seeks to inform efforts such as those of the United Nations to assess global soil erosion and inform decision makers developing national strategies for soil conservation.

## Significance

We use the latest projections of climate and land use change to assess potential global soil erosion rates by water to address policy questions; working toward the goals of the United Nations working groups under the Inter-Governmental Technical Panel on Soils of the Global Soil Partnership. This effort will enable policy makers to explore erosion extent, identify possible hotspots, and work with stakeholders to mitigate impacts. In addition, we provide insight into the potential mitigating effects attributable to conservation agriculture and the need for more effective policy instruments for soil protection. Scientifically, the modeling framework presented adopts a series of methodological advances and standardized data to communicate with adjacent disciplines and move toward robust, reproducible, and open data science.

Contemporary societies live on a cultivated planet where agriculture covers ∼38% of the land surface (1). Humans strongly depend on the capacity of soils to sustain agricultural production and livestock, which contributes more than 95% of global food production (2). The underlying agricultural systems are at the same time major drivers of soil and environmental degradation (3, 4) and a substantial source of major biogenic greenhouse gas emissions (5). The latest United Nations (UN) report on the status of global soil resources highlights that ‘…the majority of the world’s soil resources are in only fair, poor, or very poor condition’ and stresses that soil erosion is still a major environmental and agricultural threat worldwide (6). Ploughing, unsuitable agricultural practices, combined with deforestation and overgrazing, are the main causes of human-induced soil erosion (7, 8). This triggers a series of cascading effects within the ecosystem such as nutrient loss, reduced carbon storage, declining biodiversity, and soil and ecosystem stability (9). Modeling efforts to predict the impact of climate and land use change on soils are developing but limited at the global scale. The purpose of this work is to advance our ability to predict erosion given these drivers. Although we currently limit the scope to erosion by water that excludes wind, gully, and river bank erosion, it provides a valuable resource for policy makers at scales customized to their decision-making needs.

The major anthropogenic drivers of erosion are land use and potentially climate change through a more intense hydrological cycle (10). While much research attention has focused on arable agriculture (11), in a recent article we demonstrated that seminatural systems cannot be ignored, possibly accounting for ∼half of global soil erosion by water (12). Modeling soil erosion at global scales is challenging, physical models are too data intensive and the data are sparse, therefore adopting a semiempirical approach represents the state of knowledge and a pragmatic approach to informing policy. Only two studies have been successful at attempting future global soil erosion estimates, both at coarse scale (∼50 km or greater), using old climate projections and hence, impractical for policy making intervention. The pioneering geographic information system (GIS)-based Revised Universal Soil Loss Equation (RUSLE) modeling assessment conducted by Yang et al. (13) employed future projections of climate and land use that are no longer representative of the current state-of-the-knowledge; tending to overestimate soil erosion. Similarly, the study of Ito (14) simulated the effects of land cover and climate change on soil erosion by water on a 55 km mesh (1,901 to 2,100), with implications for the carbon cycle. Since these efforts, substantial progress has been made, both in terms of land use and climate projection. Recent advancements in remote sensing, wider availability of earth observation data, and increased processing of big datasets have enabled the development of new global vegetation indices and land cover products with both higher spatial resolution and accuracy (15, 16) (*SI Appendix*, Global Land Use/Cover and Future Change). The same goes for the recent release of climate datasets, including bias-corrected climate projections of multiple bioclimatic variables (17), which through robust spatial interpolation methods allow computation of global estimates of rainfall erosivity, more closely related to rainfall intensity than rainfall volume (18, 19). In this work, three alternative scenarios (2.6, 4.5, and 8.5) are tested using the Shared Socioeconomic Pathway and Representative Concentration Pathway (SSP-RCP), greenhouse gas trajectories, and multiple General Climate Models (GCMs), at a resolution (sub km) that updates the output, making it more suitable for decision-making.

Soil erosion can be mitigated using sustainable land management techniques and suitable policy incentives. Arable land, subject to conservation agriculture (CA) worldwide, is estimated to cover 11 to 14% (current study) or ∼1.42 billion hectares globally (20). Compared to a baseline scenario without any soil conservation practices, estimates indicate an overall global soil erosion reduction of about 7.1% under conservation practices (12). The footprint that human activities have left on the world’s soils are tangible (3). Soil erosion also affects nutrient and soil carbon cycling (21). The associated losses of nutrients, such as nitrogen and phosphorus, and organic carbon (22) have shown compromising long-term effects on the local ability of certain soils to meet agricultural production and ecosystem service demands (9). Global consumption of the three main fertilizer nutrients (N, P, and K) used to maintain, or improve, soil fertility is currently growing on a yearly average by 1.5, 2.2, and 2.4%, respectively (23). This comes along with high onsite economic costs for the land users to contain the production losses (12) and important cascading offsite environmental impacts (24). The pressure on fertile soils (9) through an exacerbation of soil erosion and its environmental degradation effects (25) is further strained by 1) the growing population with an estimated peak of 9.4 billion in 2070 (26), 2) the trend toward meat-intensive diets, and 3) a global climate that tends toward a more vigorous hydrological cycle (27, 28) as discussed above. These are all issues policy teams need to deal with.

Today, policy makers emphasize the need for an evidence-based approach. This modeling effort forms part of a wider program of work coordinated through the United Nations to inform national policy makers. Soil erosion is closely linked to gross domestic product (GDP) (12), with poorer countries often experiencing the greatest impacts. Hence this effort is designed to provide a globally consistent assessment to help target policy effort. It will inform countries, especially those that are often least able to identify risk, to focus resources to mitigate degradation by erosion most efficiently and effectively. Moreover, the UN endorsed the World Overview of Conservation Approaches and Technologies (WOCAT) database (29), which contains freely available information on sustainable land management (SLM) practices. By combining the model outputs with WOCAT SLMs, the two resources give decision makers a powerful set of national scale tools to identify water erosion hotspots at appropriate scales and mitigate them locally through intervention planning. While past studies have provided insights into the possible future trends of global soil erosion by water with reference to land use and climate changes (13), they have not offered the resolution for guiding policy intervention. Unique to our study is the attempt to project future changes in soil erosion at the global level using the state-of-the-knowledge harmonized set of land use and climate scenarios developed according to the new SSP-RCP adopted by the UN Intergovernmental Panel on Climate Change (IPCC). This article is a synthesis of UN collaborative effort to tackle this important socioeconomic and environmental challenge; contributing evidence in support of the upcoming UN Decade on Ecosystem Restoration (2021 to 2030). Our hope is that this contribution, along with others, will inform and empower decision makers globally to tackle soil threats and develop concerted global and national strategies for soil monitoring, conservation, and restoration.

## Results and Discussion

Achieving the UN Sustainable Development Goals (SDGs) and the UN strategies for soil conservation (30) requires understanding of the location and magnitude of erosion at global scales, now and in the future. Here, we address this challenge, in support of decision-making, by forecasting global changes in soil erosion by water, driven by land use and climate change, until 2070. We analyze mitigation opportunities through current standards of CAand reflect on possible socioenvironmental impacts related to future changes in water erosion. We also discuss the limits of our approach, quantifying the uncertainty of our estimates.

To estimate future soil erosion rates, we feed the high-resolution (250 × 250 m) RUSLE-based (12) modeling platform Global Soil Erosion Modeling (GloSEM) (*SI Appendix*, Soil Erosion Modeling) with future projections of land use obtained from the integrated assessment model (IAM) (30) (*SI Appendix*, Global Land Use/Cover and Future Change) and climate erosivity computed following the approach used in previous continental and global scale studies (18, 31) (*SI Appendix*, Climate and Future Change). The 250 m pixel resolution reflects land cover at the field scale, capturing the emergent mosaic of land use that facilitates or mitigates erosion processes. Multiple land use and climate scenarios, following three Representative Concentration Pathways (IMAGE SSP1-RCP2.6, MESSAGE-GLOBIOM SSP2-RCP4.5, and REMIND-MAGPIE SSP5-RCP8.5, with the numbers referring to the increase in radiative forcing in W·m^−2^ by 2100 (*SI Appendix*, Representative Concentration Pathway) greenhouse gas concentration trajectories adopted by the IPCC for its Fifth Assessment Report (AR5) in 2014, are modeled. Despite some limits and uncertainties addressed in the course of the manuscript, the modeling approach allows for a first estimation of soil displacement by water erosion due to sheet and rill erosion processes (referred to as “soil erosion” in the following technical definition in *SI Appendix*, Definition of Soil Erosion). These are the dominant processes on upper hill slopes because they are responsible for the largest part of soil displacement on agricultural fields and intensively used grassland (12) and found to be a fair proxy of a wider set of soil erosion processes by water (32).

**Fig. 1.**
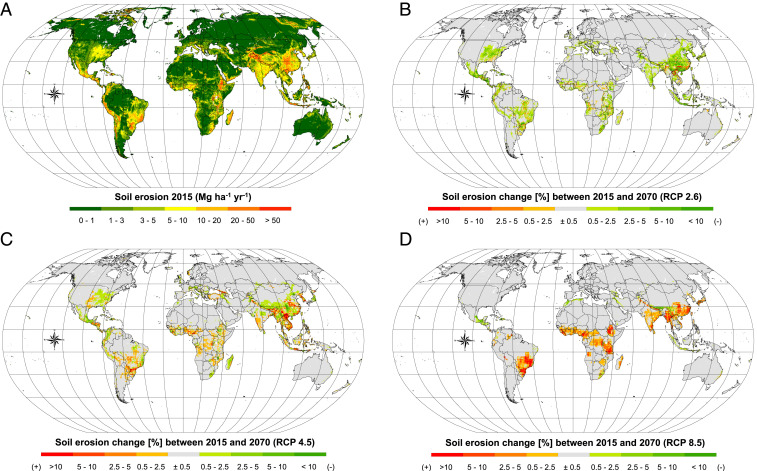
Soil erosion estimates predicted through the GloSEM. (*A*) illustrates the soil erosion rates divided into seven classes according to the European Soil Bureau classification. (*B*–*D*) illustrate changes of the annual average soil erosion between 2015 and 2070 for three distinct RCP greenhouse gas trajectories. The changes exclusively refer to effects of land use change. For these simulations, the climate of the year 2015 have been employed. (*B*–*D*) share the same legend.

What is the global pattern of soil erosion? The worldwide spatial pattern of estimated soil erosion in 2015 is illustrated in Fig. 1*A*. Our modeling results suggest that water erosion is a common phenomenon under all climatic conditions across all observed continents. However, the distribution of the spatial soil erosion patterns suggests that soil erosion seems to threaten areas of large-scale reclamation such as major agricultural sectors, especially if it occurs in conjunction with concentrated intense rainfall events (Southern Brazil, Argentina, India, East China, Midwestern United States, Ethiopia, and Mediterranean Europe). Locally, steep slopes and high-relief topography also experience high erosion rates (e.g., Andes, Himalayas, Verkhoyansk Range, and Alaska Mountain Range) together with regions with generally sparse vegetation cover across the year.

In the 2015 scenario, we estimate global soil erosion equal to $43_{-7}^{+9.2}$ Pg yr^−1^. The confidence intervals account for the uncertainty of the spatial predictions estimated using a Markov Chain Monte Carlo (MCMC) approach, the uncertainty of estimating the area under CA the uncertainty related to the effectiveness of the CA practices, and the uncertainty in regional rainfall intensity-kinetic energy relationships. The increase of about ca. 8 Pg yr^−1^ compared to previous figures reported in Borrelli et al. (12) is a consequence of the increase in the global modeling area (from ∼84.1 to ∼95.5% of the Earth’s land surface) and to a lesser extent to the land use differences derived from adopting the land use data of Hurtt et al. (30) in the current study. This estimate is lower than the figures previously presented in the scientific literature adopting similar modeling approaches, e.g., Yang et al. (13) (ca. 132 Pg yr^−1^) and Ito (14) (ca. 172 Pg yr^−1^). It supports the findings reported in the latest reference document (6) of the UN on the status of global soil resources, which indicates a value below 50 Pg yr^−1^ as a more realistic quantitative figure of global soil erosion by the processes considered (6). Insights in support of the plausibility of our modeling estimates and GloSEM limitations are reported below. We provide further information on Model Performance Evaluation and Limitations of GloSEM below and in the *SI Appendix*.

How does land use impact our estimates? Comparing soil erosion rates according to land use types, we find a decline in the estimates from croplands to forests and other forms of vegetation. Soil erosion rates are in line with field measurements (7, 33). Annual crops covered about 16% of land in 2015 and are estimated to be responsible for 41% of the total predicted soil erosion. Overall, the main agricultural lands (annual crops, permanent crops, and managed pasture) are responsible for 54% (equal to $23.4_{-4.1}^{+5.3}$ Pg yr^−1^) of the total soil erosion. Future scenarios suggest that the effects of land use change may either decrease [SSP1-RCP2.6 (Fig. 1*B*)] or increase [SSP2-RCP4.5 (Fig. 1*C*), SSP5-RCP8.5 (Fig. 1*D*)] soil erosion by 2070. The divergent trends are the result of the possible different human development and societal choices described in the three considered SSP-RCP. The IMAGE SSP1-RCP2.6 scenario, which represents a pathway aiming at limiting the increase of global mean temperature to a maximum of 2 °C by 2100, suggests a possible contraction of the main agricultural land by 2070. The simulation of this land use scenario in GloSEM yields a potential decrease in soil erosion by water of –10% (global soil erosion equal to $38.5_{-6.2}^{+8.6}$). The decrease in the simulated global soil erosion (∼4.5 Pg yr^−1^) is the result of a global reorganization of future lands, which according to the IMAGE SSP1-RCP2.6, will tend toward an overall decrease of agricultural areas in favor of an increase in forest and seminatural vegetation areas (*SI Appendix*, Fig. S1*A*). The annual crops and managed pasture are estimated to decrease globally by ca. 0.9 and 2.7 million km^2^ (equal to −2.9 and −2.4 Pg yr^−1^ of soil erosion, respectively) under the SSP1-RCP2.6 scenario, whereas the permanent crops follow a different trend with a potential increase estimated at 1.3 million km^2^ (+0.2 Pg yr^−1^). This scenario indicates a possible reduction of the share of soil erosion in agricultural land from ∼54% in 2015 to ∼48% in 2070. By contrast, the share of soil erosion in agricultural land increases to 56% and 59% under the MESSAGE-GLOBIOM SSP2-RCP4.5 and REMIND-MAGPIE SSP5-RCP8.5 scenarios, respectively. The MESSAGE-GLOBIOM SSP2-RCP4.5 is a low stabilization scenario that stabilized radiative forcing at 4.5 W/m^2^ (∼650 ppm CO_2_-equivalent) before 2100. Under the SSP2-RCP4.5 scenario, soil erosion by water would experience a slightly increased estimate at +2% (global soil erosion equal to $43.9_{-6.8}^{+9.1}$), mostly driven by the expansion of annual crops estimated at 2.1 million km^2^ (+2.6 Pg yr^−1^). This increase is partially compensated for by the resulting contraction of the managed pasture (−1.2 Pg yr^−1^) and nonagricultural lands (−0.5 Pg yr^−1^). Geographically, the SSP2-RCP4.5 shows some mixed trends (*SI Appendix*, Fig. S1*B*). Major drivers of the increase can be found in Sub-Saharan Africa, Eastern Europe, some parts of Eastern Asia, and South America. The last scenario considers very high greenhouse gases (GHG) emissions (REMIND-MAGPIE SSP5-RCP8.5) and yields a possible notable increase in future soil erosion by water of +10% (global soil erosion equal to $47.3_{-7.3}^{+9.5}$). The overall increase of soil erosion by water is estimated to be ∼4.3 Pg yr^−1^, primarily associated with substantial increases in agricultural areas in Sub-Saharan Africa, Brazil, India, Myanmar, and some districts of China. The land use conditions reported by the SSP5-RCP8.5 scenario (*SI Appendix*, Fig. S1*C*) for sizable agricultural districts in North America, Europe, and Russia would result in a lack of erosion reduction that characterizes the scenario SSP1-RCP2.6, and to a lesser extent in the SSP2-RCP4.5 scenario.

**Fig. 2.**
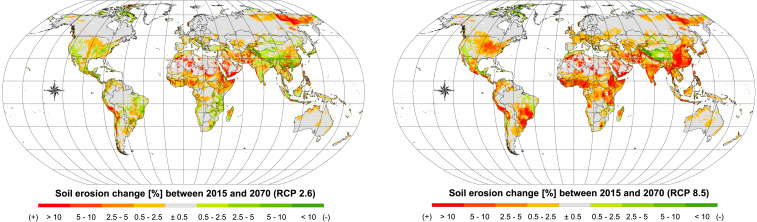
Soil erosion change between 2015 and 2070. The delta between the two observed periods [(RCP) greenhouse gas trajectories SSP1-RCP2.6 and SSP5-RCP8.5] depends on the effects of land use and land cover change and climate change.

**Fig. 3.**
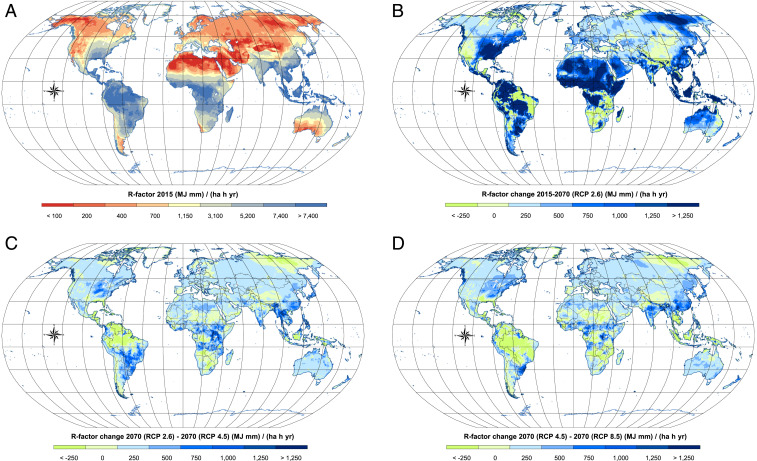
Rainfall erosivity estimates. (*A*) illustrates the global erosivity map at 30 arc-seconds (∼1 km at the equator) based on a GPR proposed by Panagos et al. (18). (*B*–*D*) illustrate changes of the annual rainfall erosivity between 2015 and the 2070 SSP1-RCP2.6 (*B*), 2070 SSP2-RCP4.5 and 2070 SSP1-RCP2.6 (*C*), and 2070 SSP5-RCP8.5 and 2070 SSP2-RCP4.5 (*D*). For the rainfall erosivity scenarios of 2070, average values of the 14 GCMs of the WorldClim database version 1.4 have been considered.

**Fig. 4.**
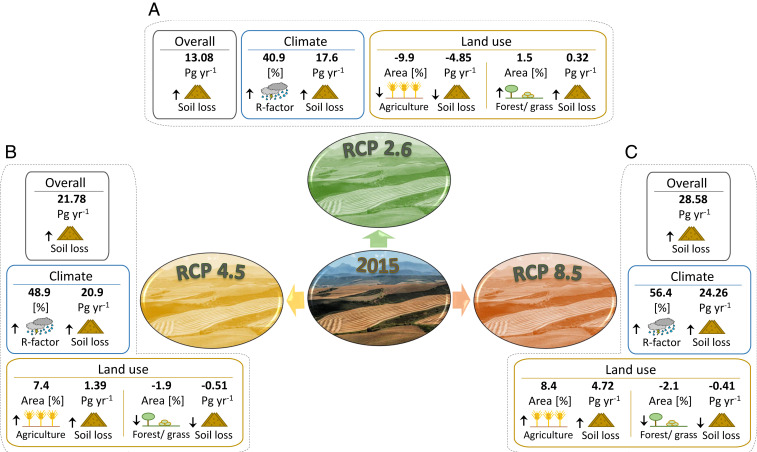
Flow diagram of the projected land use and climate changes. (*Insets*, *A*–*C*) demonstrates the projected net change of the land surface (million km^2^), climate, and soil erosion (Pg yr^−1^) for the SSP1-RCP2.6 (insert *A*), SSP2-RCP4.5 (insert *B*), and SSP5-RCP8.5 (insert *C*).

What is the combined effect of future land use and climate projections? The modeling results (Fig. 2 *A* and *B*) suggest that climate change is the major driver of the change in soil erosion. The combined land use and climate simulations show a potential substantial increase in average soil erosion totaling +30% (SSP1-RCP2.6), +51% (SSP2-RCP4.5), and +66% (SSP5-RCP8.5). Quantitatively, $56.1_{-16.4}^{+20.6}$, $64.8_{-21.4}^{+28.5}$, and $71.6_{-24.7}^{+32.5}$ Pg yr^−1^ are predicted for the SSP1-RCP2.6, SSP2-RCP4.5, and SSP5-RCP8.5 scenarios. The wider confidence intervals are related to the error propagation accounting for the variability of future climate projections of the 14 GCMs used to assess future rainfall erosivity (Fig. 3) and the set of uncertainties already considered in the 2015 scenario. These large confidence intervals associated with future climate projections reflect the higher uncertainty of these estimates. The multiscenario comparison suggests that although future land use changes can notably affect global soil erosion processes through the expansion or contraction of croplands, a global climate potentially moving toward more vigorous hydrological cycles would be acting as a major driver of future increases in soil erosion (Fig. 4). All SSP-RCP climate scenarios processed in the Gaussian process regression (GPR) model (*SI Appendix*, Climate and Future Change) indicate possible substantial increases in future rainfall erosivity following similar spatial patterns but different intensities (Fig. 2 and *SI Appendix*, Fig. S2). In absolute terms, the greatest increases occur in areas with tropical climates. Countries in temperate latitudes and with subtropical climates, however, would not be spared by increases of climate erosivity and experience increase peaks up to 50%. In these climatic zones, substantial increases might interest sectors of Eastern North America, Central and Northern Europe, Middle East, and North and East Asia. This provides important insight for policy makers identifying hotspots and trying to mitigate land degradation around the globe.

What are the key findings for policy makers? Article 1 of the UN Convention to Combat Desertification (UNCCD), ratified in March 2020 by 197 countries, identifies soil erosion as a primary cause of land degradation, which, in turn, contributes to poverty and inequality through its negative effects on agriculture, food security, and ecosystems (34). Scientific evidence suggests that global warming has already increased global economic inequality (35) and influenced immigration waves (36). The remarkable increases predicted in the combined future land use and climate change scenarios could have a wide range of negative effects at global, regional, and country levels for which this strategic analysis is intended, and not the field or catchment scale. The first and most obvious consideration emerging from this analysis is the possible exacerbation of the climatic conditions, which could substantially increase global soil erosion (+30%) already in the low GHG emission scenario (SSP1-RCP2.6). A climate-induced increase in soil erosion, as indicated by our findings, with associated land degradation and loss of key ecosystem services (9), would pose a serious threat to the achievement of a large set of targets defined by the UN strategy with the SDGs. This is particularly true for achieving 1) a land degradation neutral world by 2030 (Goal 15; Target 15.3); 2) maintaining soil quality for achieving food security (Goal 2); 3) ensuring availability and sustainable management of water resources (Goal 6); as well as 4) ending poverty (SDG 1); 5) reducing inequality (SDG 10); and 6) taking action to combat global warming (SDG 13).

How can soil erosion be mitigated? According to present knowledge, a climate-induced global increase in water erosion of such a range of magnitude could be difficult to mitigate through CA. Further runs of the GloSEM model to analyze mitigation opportunities through CA suggest that, in order to offset the most severe projected increase of global soil erosion enhanced by global land use change by 2070 (SSP5-RCP8.5, equal to $5.5_{-0.8}^{+1.1}$ Pg yr^−1^), the crops under CA would need to increase globally to ca. 60%. Current estimations indicate CA covering 11 to 14% of the global arable land area. Keeping in mind the difficulty in defining an optimal coverage of CA due to the different worldwide climate, cropping systems, and actual erosion mitigation needs and the current trends of CA, the targeted levels of CA could be missed by several European, Asian, and African countries following the current soil erosion conservation path [FAO AQUASTAT (37)]. This is considering that CA is not always exempt from shortcomings and environmental impacts itself (12). In addition, many South American and Oceanian countries already reached high percentages of cropland under CA in 2015, which limits their possible contribution to offset the projected global increases. Accordingly, if an increase of soil erosion of +10%, driven only by land use change in the SSP5-RCP8.5 scenario seems already difficult to mitigate, and would call for vigorous new conservation policies and investments, then the combined effect of future land use and climate projections modeled in the SSP5-RCP8.5 scenario may exacerbate future soil erosion processes beyond the mitigation potential of current CA standards.

How do the impacts of soil erosion propagate through the ecosystem? Maintaining soil in good health is a primary concern to farmers but the effects of soil erosion go beyond the loss of fertile land. Recognizing the link between erosion and trends of biodiversity and ecosystem service supply, the UN Intergovernmental science-policy Platform on Biodiversity and Ecosystem Services (IPBES) is calling for contributions to understand the service of sediment retention provided by natural landscapes at a global scale. Although GloSEM lacks the ability to account for deposition, on the basis of the findings provided by Grill et al. (32) we argue that increased amounts of soil eroded away from hill slopes result in increased sediment transport, which, in turn, cause ecological disturbance in the river network and reservoirs. The accumulation of these sediments in reservoirs could lead to a reduction of storage capacity and drinking water quality (SDG Goal 6: Clean Water and Sanitation). This is a challenge that already affects several low-income tropical countries that are susceptible to high levels of soil erosion and where unsafe water sources account for 6% of deaths (38). Where rivers flow into the sea, an increased deposit of sediments can exacerbate the cover of coral reefs, further compromising the existence of this fragile ecosystem (39) (SDG Goal 14: Life below water). In this regard, the spatially explicit pixel structure of GloSEM makes it a powerful tool for both mapping possible global status of soil erosion by water as well as being a course proxy indicator for sediment retention across the landscape. We observed that the present GloSEM estimates are able to explain 65% of continental and 64% of global variance in observed sediment load (32). Values reach up to more than 83% in three continents i.e., North America, Europe, and Asia. More details can be found in *SI Appendix*, Model Performance Evaluation.

**Fig. 5.**
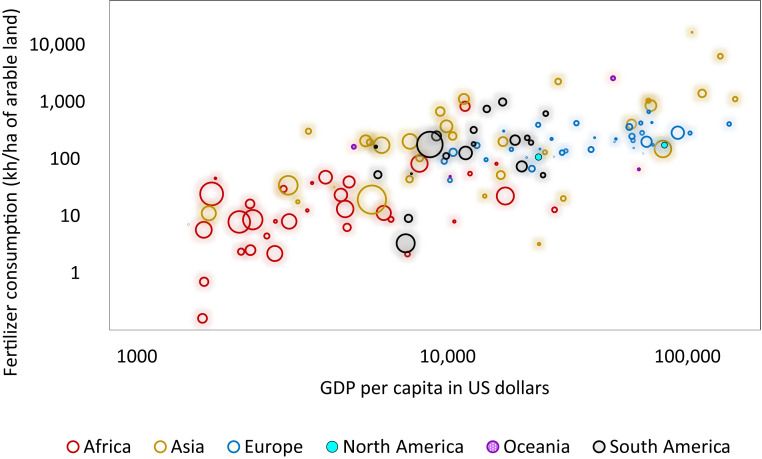
Fertilizer consumption versus GDP. Fertilizer consumption per hectare (year 2015) versus GDP) per capita (year 2011, expressed in US dollars) and the increase of soil erosion (%) between 2015 and 2070 for the SSP5-RCP 8.5. The magnitude of the increase of soil erosion is indicated by the size of the circles. The color of the circles refers to the continents.

Which economies will be impacted the most? At country level, as inferable from Fig. 2, the distribution of predicted future soil erosion patterns suggests that high-income countries, generally in temperate latitudes, may have less increase in erosion; while low- and middle-income tropical and subtropical countries may be the most susceptible to high increases of erosion. These insights are corroborated by the results presented in Fig. 5 correlating, at country level the GDP per capita, the consumption of fertilizers and the predicted increase of soil erosion between 2015 and 2070 (SSP5-RCP8.5 scenario). Except for Asia, which shows a heterogeneous situation, the other continents form visible clusters. African countries with lower GDP per capita and consumption of fertilizers in 2070 may experience the higher increase of soil erosion, with the magnitude expressed by the diameter of the circles. A similar situation can be observed for some Asian countries and to a lesser extent for some South American countries. By contrast, wealthy countries in Europe, North America, and Oceania, where levels of consumption of fertilizers are higher, show considerably lower projected future increases of erosion. Tropical countries such as Peru, Brazil, several countries in Western Africa, Cameroon, Ethiopia, Somalia, Kenya, Yemen, Southern Pakistan, India, Myanmar, Southeast China, Philippines, and Indonesia may be substantially affected by increased soil erosion. Today, roughly 2.5 billion people live in these countries. Some of sub-Saharan Africa indicates signs of considerable demographic expansion (40) with net migration flow. Future exacerbation of lands already degraded losing their ecosystem functioning and experiencing decreased agricultural productivity may aggravate the processes that displace human beings. Most of the erosion increase will occur in low-income countries that suffer from poverty and that will generally tend to be more negatively affected by climate change. To avoid the worst, it is pivotal for countries, especially in the Global South, to support the diffusion of sustainable farming practices.

It is of general interest to all countries around the world to avoid a decrease in agricultural productivity (41). According to our preliminary estimates given here, the effect of climate change will likely be so pronounced that it will overwhelm the mitigation potential of adopting soil-conserving agricultural practices. Still, without a change in agricultural practices, the effect would be multiple times worse. Countries have powerful options to positively influence their rates of soil erosion (42). Countries thus have both an incentive and a toolbox of potential measures to mitigate their soil erosion rates. Given the growing challenge of soil erosion, it is important to act now, act fast, and act comprehensively. Only by doing this through agricultural policy, interventions, and better soil governance will we ensure management to protect the health of our food production and riverine ecosystems.

### Model Performance Evaluation.

With regard to the validation of the estimates, as described in previous studies (12, 43), the validation sensu strictu of regional or larger scale applications of a model such as GloSEM is challenging due to the lack of long-term field-scale measurements; even for the present scenario simulations. The GloSEM estimates are presented with an unprecedented number of procedures to evaluate the performance of a global scale soil erosion model. This provides some highly relevant insights in support of the plausibility of the estimates of a global soil erosion model (*SI Appendix*, Model Performance Evaluation).The procedures include: 1) the assessment of the uncertainty of the spatial predictions estimated as a probability distribution through the use of Bayesian modeling; 2) the evaluation of the ability of the model to estimate soil erosion in cropland to a level close to these high-resolution regional assessments; 3) the verification that our estimates for different land use/cover units fall in the ranges of empirical measurements (metadata analysis); 4) the comparison of GloSEM soil erosion estimates with sediment transport recorded at 398 globally distributed gauging stations; 5) the evaluation of the level of agreement with expert-based and remote sensing-based UN assessments; 6) the attempt to evaluate GloSEM performance by comparing its estimates against field soil erosion measurements at plot scale; 7) the evaluation of the performance of the GPR for the present-day climate conditions; 8) the evaluation of the rainfall erosivity forecasting capacity; and 9) the assessment of the effect of every RUSLE input factor on model prediction using a sensitivity analysis.

We tested the GPR model to evaluate if it retains its prediction capability over different time frames. This is particularly important to verify our overall assumption that the GPR approach is able to perform in the temporal domain as well as in the spatial domain that we assessed previously (22). We split our data into two time sets (fitting pre-2000; validating post-2000). The results indicate a very good GPR prediction capacity for the pre-2000 training set (0.85 R^2^) and a good prediction for the post-2000 validation set (0.6 R^2^). More details are provided in the *SI Appendix*, Model Performance Evaluation.

Despite the positive insights gained through the model performance evaluation, we recognize that GloSEM, as a prediction model, founded on data-driven assumptions and a semiempirical structure, has limitations that might not allow it to capture reality fully, as is to be expected. This is particularly true for future projections which further depend on climate and land use/cover projections with higher uncertainty. The positive results highlighted by the cross-check analysis, however, suggest that the evidence reported here does represent a valuable source of preliminary information to support decision makers developing national and international strategies for soil conservation.

### Limitations of GloSEM.

As recognized by Borrelli et al. (12), and deepened in review studies (44, 45), large-scale RUSLE-based models like GloSEM that rely on data-driven assumptions have a large number of limitations. Despite the employment of state-of-the-art statistical methods, interpolation techniques, and remote sensing, GloSEM has its roots in an empirical equation like RUSLE, which heavily depends on a database that fits to the actual conditions to which it is applied. In addition, it lacks the ability to predict processes other than sheet and rill erosion. Besides the intrinsic limitations related to the RUSLE scheme, GloSEM suffers from other data-driven limitations due to the global scale at which it operates. A detailed description of GloSEM limitations are provided in *SI Appendix*, Limitations of GloSEM. This having been said, there is no alternative approach yet developed, and despite limitations the modeling provides pioneering assessment to determine potential erosion at global scales.

## Conclusions

With some degree of uncertainty, GloSEM allows prediction of both state and change of soil erosion, identifying hotspots thanks to its high resolution (250 × 250 m) and predicting future variation based on projections of change in land use, soil conservation practices, and climate change. Its estimates provide a useful knowledge base to support decision makers in considering the development of more resilient agricultural systems, such as agroforestry, regenerative agriculture, or other emerging techniques able to go beyond current CA strategies. These need to take into consideration that we may need to deal with important changes to climate now and in the coming decades. Both SSP1-RCP2.6 and SSP5-RCP8.5 scenarios provide clues on possible future local conditions requiring adaptation to drastic climate change conditions. These appear to be mainly located within highly populated tropical countries. The ability of GloSEM to identify hotspots and areas of concern at the global scale provides the basis for a more strategic approach in directing local monitoring/modeling. In addition, the dynamic nature of the model makes it suitable for both ex-ante and ex-post policy evaluation. Scientifically, the way forward for GloSEM is to produce free and easily accessible knowledge on global soil erosion dynamics to be shared with adjoining disciplines. The modeling framework presented in this study adopts standardized data in an adequate format to communicate with adjacent disciplines and moves us toward robust, reproducible, and open data science. It aims at facilitating the consideration of soil erosion processes and deriving land degradation impacts in the next assessment reports of the IPCC.

## Materials and Methods

The RUSLE-based Global Soil Erosion Modeling platform (GloSEM) (12) (*SI Appendix*, Modeling Soil Erosion by Water) was updated to establish a more comprehensive modeling framework to estimate future global soil erosion scenarios that dynamically integrate climate and land use change scenarios developed according to the new SSP-RCP adopted by the Intergovernmental Panel on Climate Change (IPCC). Here, GloSEM is combined with multiple alternative scenarios of future (2070) land use developments of the Land Use Harmonization (LUH2) project (30) (*SI Appendix*, Chapter Global Land Use/Cover and Future Change) as well as climate developments advanced in collaboration with the Joint Research Centre (JRC) of the European Commission (6, 7) (*SI Appendix*, Climate and Future Change). Standardized input data are used to allow for exchange of knowledge or possible future integration of this study’s output with those of adjacent disciplines.

### Study Area.

The study area comprises the land surface of 202 countries for which the Food and Agriculture Organization Corporate Statistical Database (FAOSTAT) (www.fao.org/faostat/en/) currently offers statistics. The modeling area totals about 143 million km^2^ (∼95.5% of the Earth’s land surface), currently providing living space for a global population of ca. 7.5 billion people and estimated to rise to about 9 billion around 2070.

### Uncertainty Analysis and Error Propagation.

A summary of error propagation in present and future predictions is calculated considering the uncertainty of the spatial predictions estimated using a MCMC, the uncertainty of estimating the area under CA (considered only for the scenario of 2015), the uncertainty related to the effectiveness of the CA practices using a Monte Carlo method approach, and the uncertainty in regional rainfall intensity-kinetic energy relationships (*SI Appendix*, Uncertainty Analysis). Further uncertainties related to the variation found in the 14 different GCMs is also considered. The error propagation is the square root of the sum of squares of the different uncertainties.

### Model Performance Evaluation.

A validation sensu strictu of a (R)USLE-type model applied at large scale is not limited by the absence of long-term field-scale measurements (12, 43). Considering that the ability of the GloSEM model to accurately measure the amount of displaced soil cannot be assessed, a cross-comparison of the modeling results to gain insights on the plausibility of the modeling predictions was performed. This was done comparing our estimate with the ones provided by other global and regional soil erosion assessments and empirical observations.

## Supplementary Material

Supplementary File

## Data Availability

All data supporting the findings of this study are available within the article text and *SI Appendix* or are freely available at the European Soil Data Centre (ESDAC), the institutional soil data repository of the European Commission Joint Research Centre (https://esdac.jrc.ec.europa.eu/themes/global-soil-erosion-future-projections) (46).
