# Supplementary material for: Land use and climate change impacts on global soil erosion by water (2015-2070)
Source: Proc Natl Acad Sci U S A. 2020 Aug 24;117(36):21994–2001. doi: 10.1073/pnas.2001403117 (PMC7486701; doi:10.1073/pnas.2001403117)
Supplement: Supplementary File [file pnas.2001403117.sapp.pdf]

PNAS
www.pnas.org

# Supplementary Information for

## Land use and climate change impacts on global soil erosion by water (2015-2070)

Pasquale Borrelli,[a,b,1], David A. Robinson,[c], Panos Panagos,[d], Emanuele Lugato,[d], Jae E. Yang,[b], Christine Alewell,[a], David Wuepper,[e], Luca Montanarella,[d], Cristiano Ballabio[d]

[a] Environmental Geosciences, University of Basel, Switzerland. [b] Department of Biological Environment, Kangwon National University, Chuncheon, Republic of Korea. [c] UK Centre for Ecology and Hydrology, Environment Centre Wales, Bangor, UK. [d] European Commission, Joint Research Centre, Ispra (VA), Italy. [e] Agricultural Economics and Policy, ETH Zurich, Zurich, Switzerland.

[1] To whom correspondence may be addressed. Email: pasquale.borrelli@kangwon.ac.kr.

**This PDF file includes:**

Supplementary text (SI Materials and Methods)

Supplementary text (SI Results and Discussion)

Figs. S1 to S15

Tables S1 to S8

References for SI reference citations



www.pnas.org/cgi/doi/10.1073/pnas.2001403117

**Supplementary Information Text**

**SI Materials and Methods**

## 1. Soil Erosion Modelling

### 1.1 RUSLE-type Models

The Revised Universal Soil Loss Equation (RUSLE) (1) (Eq. 1) is an empirical model which belongs to the detachment-limited model type. This means that although the overland flow may theoretically transport an infinite sediment amount, the quantity of sediment available to be moved is actually limited by the soil detachment capacity defined by the erosivity of the rainfall. The soil erosion (Mg $ha^{-1}$ $yr^{-1}$) resulting from sheet and rill erosion processes is given by the following multiplicative equation:

$$A = R \cdot L \cdot S \cdot K \cdot C \cdot P \quad \text{(Eq. 1)}$$

where: A (Mg $ha^{-1}$ $yr^{-1}$) is the annual average soil erosion, R (MJ mm $h^{-1}$ $ha^{-1}$ $yr^{-1}$) is the rainfall-runoff erosivity factor, K (Mg h $MJ^{-1}$ $mm^{-1}$) is the soil erodibility factor, L (dimensionless) is the slope length factor, S (dimensionless) is the slope steepness factor, C (dimensionless) is the land cover and management factor, P (dimensionless) is the soil conservation or prevention practices factor.

### 1.2 Conceptual Scheme of GloSEM

The RUSLE-based modeling platform Global Soil Erosion Modeling (GloSEM) (SI Appendix, Fig. S3) follows the same principle of most RUSLE-type models or more complex catchment-scale process-based models, with a driving force (erosivity of the climate, R), a resistance term (erodibility of the soil, K) and other factors representing the farming choice, i.e., topographical conformation of the field (LS), cropping system (C) and soil conservation practices (P). The RUSLE soil loss prediction scheme was preferred to other process-based physical models, as the latter require input data that are not yet mature enough for global scale applications. As evidenced by Borrelli et al. (2) in the assessment of the global impact of 21st century land use change on soil erosion, a simple but physically plausible empirical method for predicting soil erosion such as RUSLE can provide reasonably accurate estimates for most practical purposes (3) and policy development. This is also corroborated by the fact that all spatially explicit global estimations of soil erosion in scientific literature are currently based on (R)USLE type models (2, 4, 5, among others).

The GloSEM long-term annual soil erosion rates expressed as a mass of soil lost per unit area and time (Mg $ha^{-1}$ $yr^{-1}$) are estimated using high-resolution (ca. 250m cell size at the equator) large-scale Geographic Information System (GIS) modeling. Following a GIS raster approach means hypothesizing that each cell is independent from the others with respect to soil erosion and that the term soil erosion (or soil loss) refers to the amount of sediment that reaches the end of a specified area (cell) on a hillslope that experiences loss of soil by water erosion.

SI Appendix, Fig. S3 illustrates the workflow of GloSEM platform integrating future land use/ cover (SI Chapter 3 Global Land Use/ Cover and Future Change) and climate change (SI Chapter 4 Climate and Future Change).

### 1.3 Model Implementation

The RUSLE-based component of the GloSEM platform rests on a multiplicative equation counting six environmental factors (Eq. 1) computed as follows:

The C-factor measures the combined effect of the interrelated cover and management variables on the soil erosion process. For this global assessment, we followed the path paved in previous global studies (2, 6). Accordingly, two different approaches were undertaken to estimate C-factor values for agricultural and non-agricultural land, considering land use and its changes, the extent,

types and spatial distribution of global croplands, cropping systems and non-crop canopy cover density (SI Chapter 2 Land Cover and Management Factor (C-factor)).

The rainfall-runoff erosivity factor (R) was computed using a Gaussian Process Regression (GPR) which is explained in SI Chapter 4 Climate and Future Change.

The topographic parameter (LS-factor) representing the influence of the terrain on surface runoff and sediment transport capacity is computed through the GIS-based two-dimensional approach suggested by Desmet and Govers (7). The slope and upslope contributing area was calculated using the hole-filled SRTM and ASTER GDEM v2 data with a 3 arc-seconds spatial resolution (ca. 90m). Due to the large amount of processed data, the computational operations were carried out using the SAGA-GIS interface in the statistical computing software R. We created 870 tiles of ca. 500 x 500km. The 90m LS data was then resampled to ca. 250x250m spatial resolution applying the nearest resampling method in ArcGIS.

To spatially define the susceptibility of the soil to be eroded (K-factor), we used the algebraic approximation reported by Wischmeier and Smith (8). The soil properties (i.e., texture, organic matter, coarse fragmentation) were downloaded from the ISRIC SoilGrids database at a 1 km spatial resolution (9). Further soil properties such as soil structure and permeability were derived according to the methodology proposed for the soil erodibility map of GloSEM (2).

During the run of the baseline scenarios, the P-factor was assumed to remain constant at 1 (no support practices). The computation of the impact of the support practices on the average annual erosion rate is described in SI Chapter 7.3. Uncertainty Associated to the Effectiveness of CA Based on Topographic Conditions.

## 2. Land Cover and Management Factor (C-factor)

*C-factor for the arable land*. We used twelve-years of crop harvested area to statistically describe the crop rotations (2001-2012) of each country, while a set of 125 crops was considered. The statistical data were collected at national level from the Food and Agriculture Organization's (FAO) FAOSTAT database (http://www.fao.org/faostat/en/#data). In the following stage of data processing the 125 crops were categorized into thirteen crop groups according to their soil cover effectiveness (SI Appendix, Table S1). We assigned C-factor values to each of the thirteen crop groups according to literature thresholds (6, 10, 11) (SI Appendix, Table S2) to statistically describe typical crop rotations in each country.

In the following stage of data processing, the C-factor values ($C_{CROP}$) for each of the considered 3,252 sub-national administrative units of the 202 countries (12) were estimated as follows:

$$C_{CROP} = \sum_{n=1}^{13} C_{CROPn} \cdot [\%]Region_{CROPn} \qquad \text{(Eq. 2)}$$

where $C_{CROP}$ represents the C-factor of the n-crop and [%] $Region_{CROPn}$ represents the share of this crop in the arable land of the given region. The shares (%) of the regional crops for the rescaling operation were assessed through a statistical downscaling of the national crop statistics using the harvested areas proposed by Monfreda *et al*. (13) combining national, state, and county level census statistics with remote sensing data (for 15,990 administrative units) (14).

*C-factor for the non-arable land*. For the eleven classes of International Geosphere Biosphere Programme (IGBP) classes which reported natural vegetation, the C-factor values were defined at pixel-level through a semi-qualitative approach combining a constraining range of C-factor values obtained from literature (SI Appendix, Table S3) with a sub-pixel-level annual representation of the

average surface vegetation cover. Accordingly, the impact of the vegetation cover in the C-factor estimations was quantified by a proxy vegetation layer obtained from MODIS imagery. The surface vegetation patterns were globally described by means of the MOD44B Vegetation Continuous Fields product (VCF). This is a ca. 250m spatial resolution biophysical parameter derived from the MODIS satellite. It reports annual estimates of the percentages of i) surface vegetation cover, ii) bare soil and iii) tree cover. Data were downloaded via the NASA EarthData facility and pre-processed using the MODIS Reprojection Tool. Multiple annual data were downloaded and averaged to limit the effects of rainfall inter-annual variability on NDVI and the cloudiness variability. For the reference year 2015, the average VCF values of the years 2014, 2015 and 2016 were used. A preliminary land cover and management C-factor for non-arable land ($C_P$) was calculated as:

$$C_P = MIN_C + \left((MAX_C - MIN_C) \cdot NVS\right)$$

(Eq. 3)

where the $MIN_C$ and $MAX_C$ values were set following the literature (6, 8, 15–20). NVS (Non Vegetated Surface) was normalized to a range from 0 to 1 and described the percentage of ground covered by any vegetation type. Within a next step, the final land cover and management C-factor for non-arable land ($C_{NA}$) was computed including the tree coverage (TC) which was normalized ranging from 0 to 1:

$$C_{NA} = MIN_{CF} + \left((MAX_{CF} - MIN_{CF}) \cdot TC\right)$$

(Eq. 4)

where the $MINC_F$ and $MAX_{CF}$ values were set according to Wischmeier and Smith(10) as 0.0001 (100% canopy cover) and 0.009 (spare forest vegetation). From this data processing step, the forestland class was excluded to avoid redundancy.

## 3. Global Land Use/ Cover and Future Change

### 3.1 The Land-Use Harmonization (LUH2) Database

The Land-Use Harmonization (LUH2) (http://luh.umd.edu/) provides harmonized land use/ cover scenarios that aim to connect the historical reconstructions of land use with the future projections in the format required for Earth System Models (ESMs). The land use/ cover harmonization strategy estimates the fractional land-use patterns, underlying land-use transitions, and key agricultural management information on an annual basis, for the time period 850-2100 at 0.25° x 0.25° resolution (ca. 50 km at the equator).

### 3.2 Probabilistic Land Use/ Cover Allocation Scheme

In all scenarios (2015 and 2070) the definition of land use/ cover at 250m spatial resolution followed a probabilistic land use/ cover allocation scheme to ensure consistency between the 250m GloSEM input data and the land use/ cover conditions reported in the Land Use Harmonization (LUH2) at 0.25° x 0.25° resolution.

The land use/ cover in 2015 relies on the update of the global land use/ cover map of 2012 elaborated by Borrelli et al. (2). The allocation scheme ensured that the 250m GloSEM input data were fully consistent with the land use/ cover information reported in each 0.25° x 0.25° cell of the LUH2 database for 2015. The new 250m cell size map of 2015 was defined adopting remote sensing data of NASA's Moderate Resolution Imaging Spectroradiometer (MODIS) and a geo-statistical approach to define the extent, types, spatial distribution of global agricultural land and natural vegetation. The cells of the global land use/ cover map of 2012 to be replaced in the new version for 2015 were selected using auxiliary information provided in the MODIS MCD12Q1 product (i.e., classification confidence and second most likely IGBP class at each cell). This allowed us to perform a targeted replacement based on the classification confidence of each pixel and to

downscale the 0.25° x 0.25° resolution LUH2 to 250m using a high likelihood classification. The reallocation of arable land only needed minor local adjustments because both GloSEM and LUH2 are significantly related to recent FAO statistics (http://www.fao.org/faostat/en/).

The classification rules are described here in detail. The reallocation of managed pasture and perennial crops was done based on classification rules applied to the auxiliary information provided in the MODIS MCD12Q1 product (i.e., classification confidence and second most likely IGBP class at each cell) and Hansen's et al. (21) global forest tree cover based on Landsat imagery. In the case of managed pasture, the pixels were preferentially reclassified according the following rules: IGBP-12 rather than IGBP-14, higher classification confidence, and from lowest (0) to highest (100) wood cover. With regard to perennial crops, applied rules are IGBP-14 rather than IGBP-12 thus resulting in a higher classification confidence to select from the highest (set to 49%, non-forest land) to the lowest wood cover. This simplification of the complexity and heterogeneity of the global agricultural areas allowed us to perform a targeted selection of the areas potentially exploited as perennial crops and managed pasture.

For the 2070 scenarios, the definition of land use/ cover at a 250m spatial resolution follows a similar probabilistic land use/ cover allocation scheme, designed to spatially downscale the three selected future land use/ cover scenarios of the Integrated Assessment Model provided in the Land Use Harmonization database (LUH2), i.e., SSP1-RCP2.6 derived from IMAGE; SSP2-RCP4.5 derived from MESSAGE-GLOBIOM; SSP5-RCP8.5 derived from REMIND-MAGPIE (SI Appendix, Fig. S2). enable us to use satellite data as auxiliary information, a modified version of the projections of the FAO's Crop Suitability Index (CSI) (GAEZ, http://www.fao.org/nr/gaez/about-data-portal/agricultural-suitability-and-potential-yields/it/) was selected to act as auxiliary information for the allocation of the future land use/ cover change. More precisely, three CSIs indices (resampled to 250m using nearest neighbour resampling method) corresponding to the SSP1-RCP2.6, SSP2-RCP4.5 and SSP5-RCP8.5 scenarios were employed. These were combined with maps (250m cell size) representing the slope gradient map and cropland proximity (250m, based on the cropland of 2015 and Euclidean distance analysis performed in ArcGIS 10.5). Within a first step the three global datasets were normalized using fuzzy membership functions. Accordingly, sensitivity values ranging from 0 to 1 were assigned on a pixel basis (where 0 represents no sensitivity and 1 represents maximum sensitivity). Subsequently, for each future scenario a modified CSI index was computed through a weighted average, considering its respective original projection of CSI index (weight = 0.25), slope gradient (weight = 0.5) and cropland proximity (weight = 0.25). The reallocation of land use/ cover units was performed based on the values of the modified CSI index. In case of arable land and permanent crops, the pixels were preferentially reclassified according the following rules: non-agricultural areas in 2015 and high values of modified CSI index (i.e., higher crop suitability). Other land use/ cover pixels were allocated to areas with low values of modified CSI index according to the following order of preference: pasture, semi-natural vegetation and forest. Accordingly, forests (followed by semi-natural areas) tend to be allocated in the less favourable areas such as steeper slopes with lower crop suitability and generally not in the proximity of arable lands.

## 4. Climate and Future Change

### 4.1 Present Rainfall Erosivity Assessment

The current exposure of the Earth's surface to the energetic input of rainfall is assessed using data elaborated by Panagos et al. (22) that were already employed in previous versions of GloSEM (2). Given the good correlation between rainfall-runoff erosivity (R) and monthly climate data under current global climate conditions (22) (i.e., $R^2$ = 0.722, Root Mean Square Error (RMSE) = 1,629 MJ mm $ha^{-1}$ $h^{-1}$ $yr^{-1}$), a regression approach was used to deduce the spatial global distribution of rainfall erosivity under future climate projections (SI Chapter 4.2 Projections of Future Rainfall Erosivity). The method employed to estimate future (2070) rainfall erosivity values derives from previous studies used to estimate the future rainfall-runoff erosivity in Europe (23).

### 4.2 Projections of Future Rainfall Erosivity

Future climate projections naturally lack high-resolution rainfall data (<1 h). Accordingly, a rigorous estimation of rainfall-runoff erosivity (R) is not feasible. To estimate plausible future rainfall erosivity dynamics an approach proposed by Panagos et al. (23) to estimate future rainfall erosivity at a pan-European scale was used. Given the strong correlation of rainfall erosivity and precipitation dynamics such as precipitation seasonality and monthly precipitation (24), a regression approach was used to derive the distribution of rainfall erosivity in 2070 (dependent variable) from a series of related, but independent, WorldClim climatic variables (SI Appendix, Table S4) (SI Appendix, Fig. S4). This was done by fitting a regression model using baseline climate conditions derived from the WorldClim dataset and the rainfall erosivity as calculated from field measurements. The Gaussian Process Regression (GPR) model established a statistical relation between the R-factor point values (3,625 meteorological stations of GloREDa stations spread across 63 countries; sub-hourly pluviograph data = 61%; hourly pluviograph data = 39%) and WorldClim baseline climatic data acting as a set of spatially exhaustive covariates (fitting part in SI Appendix, Fig. S4a). In a second step, this GPR regression model was applied to WorldClim future climatic data layers for the year 2070 (for each of the fourteen General Climate Models (GCMs) and each of the three Shared Socioeconomic Pathway and Representative Concentration Pathways ((SSP-RCP), a total of 43 possible future scenarios) in order to derive the future predictions of the rainfall erosivity (R2050) (prediction part in SI Appendix, Fig. S4b).

The rationale behind this procedure rests on the assumption that rainfall intensities, and as such rainfall erosivity, are associated with given combinations of climate conditions that occur in the present. It is assumed that in the future, similar combinations of climate conditions will be related to rainfall intensities and rainfall erosivity in the same way but will likely occur at different latitudes or in different periods of the year. Consequently, applying the regression model fitted on current climate dataset allows us to estimate future levels of rainfall erosivity when the same model is applied with covariates of projected future climatic data sets.

The optimization of the GPR by feature selection was performed using a Simulated Annealing (SA) approach (25). Simulated Annealing (SA) is an optimization technique processing arbitrary degrees of nonlinearities (and stochasticity) and guarantees finding the statistically optimal solution (26). Further, SA allows finding the best set of covariates to be included in the GPR model by optimizing a chosen model metric; in this case the metric is cross-validation Root Mean Square Error (RMSE).

For the first fold, $n$-1 of the data is used in the search while the remaining $n$-($n$-1) is used to estimate the internal performance. The fitted model is then applied to all the data in order to obtain the external performance. This allows us to have two metrics, one used for fitting the model (internal performance) and the other used to express global model performance. SA also allows us to estimate variable importance by ranking variable frequency candidate models through the optimization process and their influence on the final model. Finally, the GPR equation together with the projected changes of the same covariates are used to estimate the R-factor in 2070.

The GPR could potentially use 42 covariates from the WorldClim database. These WorldClim climate data represent long-term average conditions projected for the period 2061-2080 (https://www.worldclim.org/). The fourteen (SI Appendix, Table S5) GCMs considered are part of the IPPC5 global climate data downscaled at 30 seconds resolution provided in the WorldClim. The variables used as covariates (www.worldclim.org) for the present and future interpolations (https://www.worldclim.org/bioclim) are (SI Appendix, Table S4): i) average monthly precipitation, ii) average minimum and maximum monthly precipitation, iii) average monthly temperature, iv) precipitation of the wettest month, v) precipitation of the driest month, and vi) precipitation seasonality.

### 4.3 Evaluation of the GPR Forecasting Capacity

The assumption, so far made, is that the GPR model is capable of predicting the values of rainfall erosivity (R) given a set of bioclimatic variables and that this capability is retained when making predictions across different time horizons. This assumption rests on the idea that the spatial variation of bioclimatic variables results in specific climatic conditions corresponding to specific R values. Given that GPR is capable of modelling this variation in the spatial domain (22) the same capacity can be assumed for the time domain. This assumption was tested by splitting the GloREDa time series into two datasets with one of the R values derived from meteorological series measured before 2000 (starting in 1969) and the other one measured after 2000. Using the year 2000 as the splitting point, is based on data (allows to derive two datasets with a similar number of stations) as well as climatic considerations (year 2000 marks the beginning of more dramatic climatic changes due to global warming). The latter aspect finds support in the rapid and continuously raising trend in annual global temperature anomaly highlighted by the Warming Stripes for GLOBE from 1850-2019 (Ed Hawkins - https://showyourstripes.info/). The two datasets are only partially overlapping, as only 578 stations have time series covering the two periods, while the pre-2000 dataset constitutes of 1,578 stations and the post-2000 dataset consists of 2,500 stations. Bioclimatic variables were derived from the WorldClim historical climate data, containing annual climate data from 1960 to 2018 at a spatial scale of 25km.

In order to test GPR forecasting capacity, a model was fitted on the pre-2000 data using the estimated R derived from measured data (dependent variable) and WorldClim historical data (averaged over the same timespan). The fitted GPR model was subsequently used to predict the values for the post-2000 period using the WorldClim data for the same time horizon. This procedure constitutes a form of external validation in which the validation dataset belongs to a different period.

### 5. Shared Socioeconomic Pathway and Representative Concentration Pathway

For 2015-2070, global land use conditions based on three Shared Socioeconomic and Representative Concentration (SSP-RCP) pathways were selected (SI Appendix, Fig. S5), namely SSP1-RCP2.6, SSP2-RCP4.5 and SSP5-RCP8.5. They describe different possible climatic futures scenarios depending on how much greenhouse gas will be emitted until 2070 (27) (O'Neill et al. 2016). These RCPs are labelled according to a possible range of radiative forcing values in the year 2100 (2.6, 4.5, 6.0, and 8.5 W/m2, respectively) (https://ar5-syr.ipcc.ch/topic_futurechanges.php). The original data elaborated by Hurtt et al. (28) can be downloaded at:

https://luh.umd.edu/data.shtml

SSP1-RCP2.6 scenario forms the low end of the scenario literature with regard to emissions and radiative forcing. It represents a very low greenhouse gas (GHG) emission pathway therefore limiting climate change to less than 2°C by 2100. Also known as the sustainable scenario, it assumes that 'the world shifts gradually, but pervasively, toward a more sustainable path, emphasizing more inclusive development that respects perceived environmental boundaries. Driven by an increasing commitment to achieving development goals' (27). IMAGE SSSP1-RCP2.6 scenario is simulated by IMAGE 3.0 integrated assessment model (29). The model framework describes i) future agriculture system, ii) changes in land cover, iii) energy system, iv) hydrological cycle, v) carbon cycle, and vi) climate change. With regard to population, IMAGE SSP1-RCP2.6 scenario (28) is characterized by moderate population growth leveling off by mid-century, and by high economic growth and technological improvements including agricultural productivity. IMAGE 3.0 data and documents can be accessed at:

https://models.pbl.nl/image/index.php/Welcome_to_IMAGE_3.0_Documentation

RCP 4.5 scenario can be considered as the 'middle of the road' scenario, where the historical patterns of development are maintained throughout the 21st century. It assumes the stabilization of the radiative forcing at 4.5 W $m^{-2}$ before 2100 without exceeding that value. The population is forecasted to peak between 2070-2080 to about 9.5 billion. This constitutes a lower value than the

U.N. medium fertility scenario of ca. 11.5 billion. The scenario is simulated in GloSEM using the data derived from MESSAGE-GLOBIOM SSP2-RCP4.5. MESSAGE is a modelling framework for medium- to long-term energy system planning, energy policy analysis, and scenario development used in conjunction with other models such as Global Biosphere Management Model (GLOBIOM), which includes a bottom-up representation of the agricultural, forestry and bio-energy sector. As reported by Hurtt et al. (28), an important feature of SSP2-RCP4.5 is the initial decrease in forest by about 43 million ha from 2000 to 2050 (comparable to the reference scenario), with a subsequent increase in forest by about 331 million ha from 2050 to 2100. MESSAGE data and documents are provided at:

https://iiasa.ac.at/web/home/research/researchPrograms/Energy/MESSAGE.en.html

SSP5-RCP 8.5 scenario forms the upper end of the scenario literature in terms of emission and radiative forcing. Also known as the 'fossil-fueled development' (27), 'this world places increasing faith in competitive markets, innovation and participatory societies to produce rapid technological progress and development of human capital as the path to sustainable development. Global markets are increasingly integrated.' This emission pathway assumes the possibility to reach a radiative forcing of 8.5 $Wm^{-2}$ in 2100 as a result of very high levels of fossil fuel employment, almost doubling of global food demand, and a greenhouse gas emission increase of up to 300% over the century. The population is forecasted to peak and decline in the 21st century. This scenario is simulated in GloSEM using the data derived from REMIND-MAGPIE SSP5-RCP8.5. It is an integrated assessment modeling platform coupling the REMIND (Regionalized Model of Investment and Development) and the MAGPIE (Model of Agricultural Production and its Impacts on the Environment) models. As reported by Hurtt et al. (28), this SSP5-RCP8.5 scenario predicts a remarkable expansion (300 Mha, equal to ~20%) of global cropland (mostly into pasture and forest) between 2010 and 2100 in response to the projected strong increase of food and feed demand. REMIND data and documents are provided at:

https://www.pik-potsdam.de/research/transformation-pathways/models/remind

## 6. Definition of Soil Erosion

In this study the term soil erosion refers to RUSLE soil loss (i.e., an estimate of the soil loss due to sheet and rill erosion processes). The RUSLE '*Soil loss refers to the amount of sediment that reaches the end of a specified area on a hillslope that is experiencing net loss of soil by water erosion. It is expressed as a mass of soil lost per unit area and time. There are several aspects of erosion that are implied in this definition. First of all soil loss refers to net loss, and it does not in any way include areas of the slope that experience net deposition over the long-term. As such, soil loss does not equate to the sediment yield from a hillslope that exhibits toe-slope deposition, which are most cases. It is, rather, the sediment delivered to the bottom of the slope area that feeds onto the toe slope*' (30).

## 7. Uncertainty Analysis

### 7.1 Uncertainty of the Spatial Predictions

RUSLE is a purely deterministic model in which the product of physical measures is used to derive the amount of soil loss. As such, a rigorous assessment of uncertainties is not feasible. In this study, we follow an approach that represents uncertainty as a probability distribution using a Bayesian modeling technique. The idea is to use the data distribution to estimate the uncertainty of the prediction. Given that RUSLE is based on the product, to simplify, all layers were log-transformed. Within a next step, each of the input layers was treated as a spatial random field. A random field is a stochastic process defined in terms of expectation and covariance. Once these two parameters are estimated, different simulations for each field can be created. Each simulation has the same parameters, but differs due to the stochasticity of the process. By combining a large number of simulations, one could, in principle, estimate how the uncertainty propagates to the model output (soil loss). As deriving spatially continuous simulations for each of the layers is

impractical, a simulation approach based on Gibbs sampling and an additive model was used. The model is expressed as:

$$z\ (S_0) = z(R) + z(LS) + z(K) + z(C) + e(s) \qquad \text{(Eq. 5)}$$

where the z() values are realizations from each of the log-transformed model input layers and e(s) is the spatial component of the model. A Markov Chain Monte Carlo (MCMC) algorithm was used to derive realizations of $z(S_0)$ (soil loss) by simulating Vb from the multivariate normal distribution with zero mean and covariance matrix, where Vb is the Bayesian covariance matrix of the fitted model. MCMC was applied using the JAGS software (31) through R interface (32).

### 7.2 Uncertainty of the Conservation Agriculture Application

The modeling estimates considering the possible mitigation effects of soil conservation rest on the Conservation agriculture (CA) data provided by 54 countries to FAO/AQUASTAT. These countries regularly communicated the proportion of their cropland managed in accordance with the three FAO CA standards (i.e., minimum soil disturbance, organic soil cover, crop rotation/ association). Overall, these countries cover 73% of the global cropland. For the remaining 27% of global croplands, the continental level of CA is considered. The lack of this information is compensated in the modeling result arbitrarily assuming an uncertainty of the estimates in the arable land of about ± 10%. In addition, FAO/AQUASTAT only provides the CA data as a percentage of the national arable land. Accordingly, no spatial information about the application of CA is available. To reconstruct the possible effect of the soil conservation practices, maintaining the link with the national topography conditions, climate and soil properties, we randomly simulated 250 CA scenarios for each country. In each run, the spatial distribution of areas under CA, the possible effectiveness of CA according to topographic conditions (SI Chapter 7.3) and the effectiveness range related to their application (i.e., application effectiveness, ranging from 75 to 100% in soil loss reduction) were randomly simulated.

### 7.3. Uncertainty Associated to the Effectiveness of CA Based on Topographic Conditions

To reconstruct the possible effect of the soil conservation practices, maintaining the link with the national topographic conditions, climate and soil properties, we randomly simulated 250 conservation agriculture (CA) scenarios for each country. In each run, for the national area under CA the possible effectiveness of CA according to topographic conditions (ranging from 45 to 75% compared to conventional tillage, consistent with the limits proposed by Borrelli et al. (2)) and an additional effectiveness range related to efficiency of the application of the CA techniques (application effectiveness, from 75 to 100% of the maximum CA reduction calculated for the specific pixel) were randomly simulated. The simulation was performed using R statistics (SI Appendix, Fig. S6), where the percentage of the national arable land under conservation was combined with three spatial sets of information (250m cell size raster data): i) a raster reporting the national arable land, ii) a raster reporting the annual average soil erosion rate estimated for the 2015 baseline scenario (no CA), and iii) a raster reporting the potential soil loss reduction due to the application of CA techniques in each pixel. The latter was estimated applying a reclassification role (SI Appendix, Table S6) to a soil slope gradient raster derived from the hole-filled SRTM and ASTER GDEM v2 data with a 3 arc-seconds spatial resolution (ca. 90m). The results of the 250-conservation agriculture (CA) simulations were used to estimate possible errors associated with the unknown spatial distribution of the arable land under CA for each country. Relevant statistics were calculated from the 250 CA random simulations. The confidence intervals were computed with the standard error method (confidence level 95%).

### 7.4 Uncertainty of the Rainfall Intensity–kinetic Energy Relationship

The average annual rainfall-runoff erosivity R-factor values (MJ mm $ha^{-1}$ $h^{-1}$ $yr^{-1}$) for all the GloREDa's meteorological stations were computed by Panagos et al. (22) following the RUSLE handbook instructions. Accordingly, the energy of a rainstorm event, which is a function of the

amount of rain and the rainstorm's intensity components, was globally calculated using the equation proposed by Brown and Foster (33):

$$E = e_{max} \cdot \left[1 - a\, e^{(-b\, I_r)}\right] \quad \text{(Eq. 6)}$$

where E denotes the rainfall kinetic energy of 1 mm of the rain expressed in MJ $ha^{-1}$ $mm^{-1}$ and $I_r$ as the rainfall intensity expressed in mm $h^{-1}$, while $e_{max}$ denotes maximum kinetic energy contents and *a* and *b* are empirical constants. In the case of Brown and Foster (33) $e_{max}$ is 0.29 based on the work conducted in Australia by Rosewell et al. (34), while the values of *a* and *b* obtained using rainfall data in US are 0.72 and -0.05, respectively.

In order to account for the rainfall intensity–kinetic energy (KE-I) relationships for different world regions, we use the coefficients of the exponential relationships ($e_{max}$, *a* and *b* in Eq. 6) fitted through field experiments reported in literature (30, 35). This procedure is similar to the one van Dijk et al. (35) used to derive their KE-I universal relationship. We used regional KE-I relationships and their standard deviations to quantify KE-I uncertainty in the GloSEM estimates. The produced energy estimates are in the range of the relationships Brown and Foster (33) used in GloSEM (14%).

### 7.5 Error Propagation

A summary of error propagation in present and future predictions was calculated considering the uncertainty of the spatial predictions estimated using a Markov Chain Monte Carlo (MCMC) approach, the uncertainty of estimating the area under conservation agriculture (CA) (considered only for the scenario of 2015), the uncertainty related to the effectiveness of the CA practices using a Monte Carlo method, and the uncertainty in regional rainfall intensity-kinetic energy relationships. Further uncertainty related to the variation found in the fourteen different General Circulation Models (GCMs) is also considered. The error propagation is the square root of the sum of squares of the different uncertainties.

## SI Results and Discussion

### 8. Model Performance Evaluation

As described in previous studies (2, 36) the validation *sensu strictu* of regional or larger scale applications of a model such as GloSEM is challenging due to the lack of long-term field-scale measurements; even for the present scenario simulations. Therefore, GloSEM is presented with a set of procedures to gain insights to support the plausibility of the global estimates.

i. **The uncertainty of the spatial predictions**. The uncertainty of the spatial predictions is estimated using a probability distribution based on Bayesian modeling techniques (i.e., Markov Chain Monte Carlo (MCMC) approach) using the data distribution to estimate the uncertainty in the modelling prediction (15). The error of the model estimates associated with the input data assessed via the MCMC approach is about 8 Pg $yr^{-1}$ for the whole world (reference year 2015). This was computed calculating sample quantiles on the simulated data. Note that the error interval is not symmetric around the average value. Accordingly, the lower error limit (at 0.9 CI) is ca. 2.4 Pg $yr^{-1}$, while the upper is narrower 5.6 Pg $yr^{-1}$. The spatial uncertainty is shown in SI Appendix, Fig. S7 as the standard deviation of the MCMC simulated values. The map provides an outline of the spatial global distribution of the estimated variance, which is used to associate the potential error across the different regions of the globe.

ii. **GloSEM cross comparison with continental studies**. A first cross-comparison of the GloSEM results was performed comparing aggregated regional values of predicted soil erosion against the values obtained by regional soil erosion assessments. The goal of this

first analysis was to assess the plausibility of the quantitative soil erosion estimates. The first regional comparison was carried out for the cropland of the United States. According to the National Resources Inventory (NRI) of the US Department of Agriculture (37), the soil loss due to sheet and rill erosion in US croplands is estimated to be 1.59 in 1982 and 0.96 Pg $yr^{-1}$ in 2012. GloSEM estimates for soil erosion in US croplands during the same periods (considering the annual conditions of land use and conservation agriculture) are equal to 1.52 and 0.91 Pg $yr^{-1}$ in 1982 and 2012, respectively. The deviation between the statistical data of the USDA and our model estimates is in both cases below 5%. The second regional comparison was carried out for cropland of the European Union using the higher spatial resolution (25m) RUSLE-based model developed by the European Commission (38). Here, the comparison also reveals a consistency of the soil erosion estimates for the European cropland in the selected reference year 2012 (GloSEM = 0.304 Pg $yr^{-1}$; EC RUSLE2015 = 0.30 Pg $yr^{-1}$).

The minor deviations (both below 5%) of the GloSEM estimates (full details in Borrelli et al. (2)) with those provided by independent studies of the European Commission and US Department of Agriculture provides first evidence of the plausibility of our quantitative estimates. It also shows that they are reliable and valid to a level close to these higher resolution regional assessments. It is important to bear in mind that measurements are also not without error, both for the measurement itself and the sampling. Thus we believe our estimates of model error to be as appropriate and rigorous as those for comparable measurement error.

iii. **GloSEM comparison with empirical data**. Further support for the plausibility of the model predictions comes from the comparison of our results with empirical data resulting from the most extensive collection of soil erosion data reported by Montgomery (39). Adapting Montgomery's (39) graphical illustration (SI Appendix, Fig. S8), we could superimpose the results from our global analysis for different land covers, to which we have added data on native forests and data from other meta-analysis studies. This analysis at meta-data level confirms that our estimates for different land use/ cover units fall in the ranges of empirical measurements. Our modelling results tend to form a lower bound for measured values on cropland and the highlands, mid-range for the bare soil areas and forests, and an upper bound for cratons. The lower bounds for the cropland and highlands could be due to study bias, with erosion measurement studies tending to be reported for areas with erosion problems or even on erosion hot spots (40).

iv. **GloSEM sediments comparison with river sediments load**. A further element in favor of the plausibility, as well as the broader utility, of the GloSEM estimates came from the comparison between the potential sediment load (PSL) derived from GloSEM estimates with values of river sediment load. Considering that river sediment loads are driven by sediment supply, in Grill et al. (41) we used GloSEM estimates as a proxy to calculate the sediment supply to global rivers. This analysis rests on the assumptions that the spatially explicit estimates of soil displacement due to sheet and rill erosion estimated by GloSEM, despite neglecting deposition, denudation and fluvial conveyance processes, can represent an indicator to quantify the potential spatial variability in sediment supply within basins. To test the quality of Grill's et al. (41) global sediment model results, we compared the PSL derived from GloSEM estimates (computed in the river system at each river reach globally) against sediment transport observations recorded at 398 gauging stations globally (42–45) (SI Appendix, Fig. S9a). We observed that the GloSEM estimates were able to explain 64% of global and 65% of continental variance in observed sediment load and more than 83% for three continents (North America, Asia and Europe) (SI Appendix, Fig. S9b-c). Within seven

basins for which multiple observations were available (i.e., Amazon, Blue Nile, Irrawaddy, Mekong, Niger, Salween and Red River), we found that the modelled PSL explains on average 81% of the observed intra-basin variance. This indicates an acceptable performance of our global sediment model. Overall, these findings suggest that the estimates made by GloSEM offer a plausible proxy indicator for sediment origins and spatial patterns of sediment connectivity within individual river basins, which, in turn, provides further evidence for the plausibility of the soil erosion estimates.

v. **Comparison with past U.N. global assessments**. Further insights in support of the plausibility of the GloSEM global estimates were gained by comparing its spatial patterns of soil erosion with the ones reported by previous U.N. global studies on soil erosion and land degradation. The first comparison was performed against the areas classified as susceptible to water erosion in the expert-based U.N. GLASOD assessment (Global Assessment of Human-induced Soil Degradation) (46). We used a study conducted in the 1980s which combined data collected by ca. 300 scientists globally. Despite the different nature of the observed period of the two assessments under comparison, the results in the areas located between the 50°S and 50°N often indicate good to fair spatial agreement (SI Appendix, Fig. S10a). The second comparison was made against the remote sensing-based U.N. GLADA assessment (Global Assessment of Land Degradation and Improvement) (47), which employed remote sensing NPP (net primary productivity) as a proxy for describing land degradation. In this case, a good spatial agreement between the patterns of the two compared databases was also frequently noticed (SI Appendix, Fig. S10b). A good agreement between the results of GloSEM and the two independent U.N. global assessments is particularly true for Central and South America, Sub-Saharan Africa, Oceania, Eastern United States, India, Eastern Europe and Central and North Asia.

vi. **Comparison with field/plot measured data**. We also attempted to evaluate GloSEM performance by comparing its estimates against field soil erosion measurements at plot scale. To do so, we used the large database of erosion rates measured on runoff plots under natural rainfall conditions recently published by Xiong et al. (48). To the best of our knowledge, this is the most comprehensive publicly available database of measurements on soil erosion in field plots. It comprises soil loss data for 2,036 runoff plots in 27 countries, representing 11,439 plot years. The database contains a large set of information including: land use types; geographic coordinates; slope gradient, slope length, annual precipitation, soil loss, run-off, plot treatment and observed period. An *ad hoc* run of GloSEM was conducted, where for consistency the topographic LS-factor was estimated using the values of slope gradient and slope length reported in the database.

A regression analysis between the long-term average annual rates estimated by GloSEM and the annual field measurements indicated an absence of correlation (n = 4,196, $R^2$ .07, $\sigma <$ .001). This non-correlation pattern was also observed comparing measured and modelled soil erosion rates averaged by plot location, i.e., aggregating all the plots fitting within a GloSEM cell (n = 177, $R^2$ .01, $\sigma >$ .2). The absence of correlation did not come as a surprise to us given very different supports. The reason why GloSEM estimates cannot explain the variance in the plot erosion rates is due to the inability to perform a rigorous comparison between measured and modelled erosion rates. Rigorous comparison can only be achieved if GloSEM model input parameters could closely reproduce the spatial scale and the conditions (e.g. land cover, rainfall intensity) occurring during the plot measurements (49). The generic and long-term GloSEM estimates for the year 2015 could not reproduce the specific plot conditions which constitute of (a) short-term measurements (average measurements of 2 years) covering a long time span (1931-2014), (b) observed high intra-annual variability of the natural rainfall

conditions, (c) average plot size (223$m^2$) much smaller than the GloSEM spatial resolution (250x250m, equal to 62,500$m^2$), (d) lack of details about the type of crops and soil conservation practices, (e) lack of information on the status and canopy cover conditions of the natural vegetation and the eventual occurrence of human disturbances, and show (f) weak correlations when comparing Xiong et al. (42) data of observed soil loss against annual rainfall (n = 4,196, R2 .04, σ < .001), slope length (n = 4,196, R2 .0, σ < .001) and slope gradient (n = 4,196, R2 .03, σ < .001).

The comparison of the descriptive statistics (i.e., average, median and standard deviation) of measured and modeled erosion rates aggregated by land use/ cover types (i.e., crops, fallow, forest and semi-natural vegetation) revealed a stronger correlation (n = 4, $R^2$ .99, σ < .001) (SI Appendix, Table S7). These results fit to the analysis at meta-data level presented above. This reinforces the idea that our estimates for different land use/ cover units fall in the ranges of empirical measurements. Given the quantitative and harmonized nature of the GloSEM data set, the ability of the model estimates to fall in the ranges of empirical measurements for different land use/ cover units indicate a possible consistency between the estimates for the two time periods (driven by land use change) as well as the reliability of the resulting geographical trends.

vii. **Uncertainty of the climate erosivity**. The performance of the Gaussian Process Regression model to estimate the rainfall erosivity for the present-day climate conditions was tested for both, a fitting (R2 = 0.81, σ <0.001), and a cross-validation (R2 = 0.722, Root Mean Square Error = 1,629 MJ mm $ha^{-1}$ $h^{-1}$ $yr^{-1}$) dataset. The cross-validation was carried out by random sampling with 10% replacement of the original dataset used for validation. More details can be found in Panagos et al. (22) (SI Appendix, Table S8).

viii. **GPR Forecasting Capacity**. The results are shown in SI Appendix, Fig. S11 as scatterplots between observed and predicted values. SI Appendix, Fig. S11 evidences a very good prediction capacity for the pre-2000 training set (0.85 $R^2$) and a good prediction capacity for the post-2000 validation set (0.6 $R^2$). Note, however, that the model tends to be less effective with data with very high R values which mainly appears at >10,000 MJ mm $h^{-1}$ $ha^{-1}$ $yr^{-1}$. Here, the model tends to underestimate the predicted R values. Further note, that the resolution of the WorldClim data is rather coarse and this might result in a mismatch between station measured data and WorldClim data, especially in areas where the topography strongly influences precipitations. Moreover, the two datasets are spatially mismatched which means that the stations from the pre-2000 dataset do not necessarily overlap those of the post-2000 dataset. This introduces more error as the model lacks a full spatial coverage allowing it to extrapolate to more diverse climatic conditions. Nevertheless, the model shows little bias when predicting post-2000 R values, comparable to that of the training data (pre-2000).

The picture changes when the model is tested on the stations for which the timeseries covers the whole period. Here, the model has the same spatial coverage for the two datasets, thus ensuring spatial congruence. Accordingly, the performance of the fitted and the validation datasets is similar (0.85 $R^2$) as shown in SI Appendix, Fig. S12. The reduction of the residual variance supports the hypothesis that a consistent portion of the error in SI Appendix, Fig. S11 is due to the spatial mismatch of the stations and measurement bias among the different stations.

The spatial distribution of the residuals (SI Appendix, Fig. S13) shows errors lower than the R value for most of the forecasted R values. Fig. S13 shows residuals as the proportion of the observed R value:

$$Std\ residual\ =\ residual\ /\ observed\ R\ value$$

(Eq. 7)

Values between 0 and 1 (or -1) indicate errors smaller than the predicted R value. SI Appendix, Fig. S13 shows that most of the values with proportionally higher error are in desert areas (e.g., Israel, Gobi Desert), where precipitation is scarce making R estimation in these areas challenging.

Fig. S14 shows the distribution of the residuals in relation to the observed R-factor values. For both the Pre-2000 and the Post-2000 sets, residuals appear to be normally distributed with a mean close to zero. The relative error (color scale) reaches higher values for observed R-factor values close to zero, while higher values of R result in deviations around 30% of the observed value. The dispersion of the residuals appears to be similar in the training (Pre-2000) and in the test (Post-2000) sets, supporting the hypothesis that errors are of random nature and not related to the model being unable to forecast future values.

ix. **Sensitivity analysis**. The influence of GloSEM input factors on the global estimates generated by the sensitivity analysis already reported by Borrelli et al. (2) is shown in SI Appendix, Fig. S15. From this sensitivity analysis, we observed that the global RUSLE-based model was most sensitive to the cover-management factor (C-factor) and, to a lesser extent, to the climate erosivity factor (R-factor). This supports the hypothesis that future changes in soil erosion are primarily driven by land use and climate change, rather than changes in soil properties and erosivity.

Despite the positive insights gained through the model performance evaluation reported above, we recognize that GloSEM as a prediction model, founded on data-driven assumptions, has a number of limitations (see below Chapter 9 Limitations of GloSEM) that do not allow it to capture reality fully, as is to be expected. This is particularly true for future projections which further depend on climate and land use/ cover projections with higher uncertainty. That said, both present and future estimates are provided with their respective uncertainties and represent the best estimates achievable with the current state-of-the-knowledge. In light of the insights gained, we believe that despite the limitations common to all large-scale modeling exercises, the estimates provided shed new light on possible future challenges that global societies may face.

## 9. Limitations of GloSEM

GloSEM is a RUSLE-based modeling approach and as such includes universally recognized factors that affect soil erosion due to sheet and rill erosion processes (50), and as suggested by theoretical studies a simplified but logical structure to simulate the physical soil erosion process (3). A recent review study conducted by Alewell et al. (51) states that there are good reasons to employ a (R)USLE-based approach while addressing soil erosion at large-scale. This is especially true as it is one of the few models available that operate at this scale. Today's state of the art (R)USLE-type models have no higher uncertainty than more complex models. As reported by Alewell et al. (51) this does not make the model free of limitations such as: (i) no simulation of soil deposition; (ii) inability to predict the removal of soil along drainage lines due to processes such as gully erosion; (iii) limitations in the model structure that allows only limited interactions and inter-relationships between the basic multiplicative factors; (iv) in the development of the equations mostly medium-textured surface soils of the Midwestern United States (US) were used, therefore, when applied in climate regimes different from those for which it was developed in the US, a greater uncertainty might be expected for the estimates.

In the interest of completeness of intrinsic model limitations, we note further known limitations associated with upscaling to global scale and the future projections: (i) we considered a set of land use types greater and sometimes considerably different from the ones for which the RUSLE model was developed (agricultural conditions), such as forestland, rangeland and other semi-natural vegetation; (ii) as discussed by Alewell et al. (51) US soils are not unique but are still

not an unbiased sample of worldwide soils such as soil erosion in tropical soils, soils in Asia, Africa and Australia which were under-represented in the US database used to develop RUSLE; (iii) despite adopting advanced interpolation techniques to spatialize the rainfall erosivity computed on a dataset of 3,625 stations covering 63 countries, the number of stations is still not large enough and homogeneously distributed enough to compensatively cover all possible different regional patterns, particularly in some regions of Africa, South America and Asia; (iv) the use of a 3 arc-seconds (ca. 90m at the equator) Digital Elevation Model ensured the computation of the combined topographical factor (LS) maintaining a scale congruence and within the one used during the USLE's experimental measurements (less than or equal to 122m) but above the most common plot measurements (22m); and (v) we were unable to reproduce possible future effects of climate on soil erodibility in the model. A further known limitation of GloSEM (vi) is its current inability to thoroughly consider the temporarily effects of impactful land use transitions due to forest disturbance and wildfires.

**References**


1. K. Renard, G. Foster, G. Weesies, D. McCool, D. Yoder, Predicting soil erosion by water: a guide to conservation planning with the Revised Universal Soil Loss Equation (RUSLE). *Agric. Handb. No. 703*, 404 (1997).
2. P. Borrelli, *et al.*, An assessment of the global impact of 21st century land use change on soil erosion. *Nat. Commun.* **8** (2017).
3. V. Ferro, Deducing the USLE mathematical structure by dimensional analysis and self-similarity theory. *Biosyst. Eng.* **106**, 216–220 (2010).
4. J. N. Quinton, G. Govers, K. Van Oost, R. D. Bardgett, The impact of agricultural soil erosion on biogeochemical cycling. *Nat. Geosci.* **3**, 311–314 (2010).
5. K. Van Oost, *et al.*, The Impact of Agricultural Soil Erosion on the Global Carbon Cycle. *Science (80-. ).* **318**, 626–9 (2007).
6. P. Panagos, *et al.*, Estimating the soil erosion cover-management factor at the European scale. *Land use policy* **48** (2015).
7. P. Desmet, G. Govers, A GIs procedure for automatically calculating the USLE LS factor on topographically complex landscape units. *J. Soil Water Conserv.* **51**, 427–433 (1996).
8. W. Wischmeier, D. D. Smith, W. H. Wischmer, D. D. Smith, Predicting rainfall erosion losses: a guide to conservation planning. *U.S. Dep. Agric. Handb. No. 537*, 1–69 (1978).
9. T. Hengl, *et al.*, SoilGrids1km — Global Soil Information Based on Automated Mapping. *PLoS One* **9**, e105992 (2014).
10. W. H. Wischmeier, D. D. Smith, Predicting rainfall erosion losses. *Agric. Handb. no. 537*, 285–291 (1978).
11. P. Borrelli, *et al.*, Effect of Good Agricultural and Environmental Conditions on erosion and soil organic carbon balance: A national case study. *Land use policy* **50**, 408–421 (2016).
12. FAO, Global Administrative Unit Layers (GAUL) (2016) (May 15, 2016).
13. C. Monfreda, N. Ramankutty, J. A. Foley, Farming the planet: 2. Geographic distribution of crop areas, yields, physiological types, and net primary production in the year 2000. *Global Biogeochem. Cycles* **22**, 1–19 (2008).
14. N. Ramankutty, A. T. Evan, C. Monfreda, J. A. Foley, Farming the planet: 1. Geographic distribution of global agricultural lands in the year 2000. *Global Biogeochem. Cycles* **22** (2008).
15. A. J. J. Van Rompaey, G. Govers, Data quality and model complexity for regional scale soil erosion prediction. *Int. J. Geogr. Inf. Sci.* **16**, 663–680 (2002).
16. USDA, *Procedure for computing sheet and rill erosion in project areas* (U.S. Department of Agriculture, 1977).
17. D. E. Walling, The evolution of sediment source fingerprinting investigations in fluvial

systems. *J. Soils Sediments* **13**, 1658–1675 (2013).
18. D. Yang, S. Kanae, T. Oki, T. Koike, K. Musiake, Global potential soil erosion with reference to land use and climate changes. *Hydrol. Process.* **17**, 2913–2928 (2003).
19. M. Märker, *et al.*, Assessment of land degradation susceptibility by scenario analysis: A case study in Southern Tuscany, Italy. *Geomorphology* **93**, 120–129 (2008).
20. L. Borselli, P. Cassi, D. Torri, Prolegomena to sediment and flow connectivity in the landscape: A GIS and field numerical assessment. *Catena* **75**, 268–277 (2008).
21. M. C. Hansen, *et al.*, High-Resolution Global Maps of 21st-Century Forest Cover Change. *Science (80-. ).* **342**, 850–853 (2013).
22. P. Panagos, *et al.*, Global rainfall erosivity assessment based on high-temporal resolution rainfall records. *Sci. Rep.* **7**, 1–12 (2017).
23. P. Panagos, *et al.*, Towards estimates of future rainfall erosivity in Europe based on REDES and WorldClim datasets. *J. Hydrol.* **548** (2017).
24. P. Panagos, *et al.*, Monthly rainfall erosivity: Conversion factors for different time resolutions and regional assessments. *Water (Switzerland)* **8** (2016).
25. S. Kirkpatrick, C. D. Gelatt, M. P. Vecchi, Optimization by Simulated Annealing. *Science (80-. ).* **220**, 671–680 (1983).
26. L. Ingber, Simulated Annealing : Practice versus Theory. *Math. Comput. Model.* **18**, 29–57 (1993).
27. B. C. O'Neill, *et al.*, The Scenario Model Intercomparison Project (ScenarioMIP) for CMIP6. *Geosci. Model Dev.* **9**, 3461–3482 (2016).
28. G. C. Hurtt, *et al.*, Harmonization of Global Land-Use Change and Management for the Period 850-2100 (LUH2 for CMIP6. *Geosci. Model Dev. Discuss.*, 1–65 (2020).
29. E. Stehfest, *et al.*, *Integrated assessment of global environmental change with IMAGE 3.0* (2014).
30. M. A. Nearing, S.-Q. Yin, P. Borrelli, V. O. Polyakov, Rainfall erosivity: An historical review. *Catena* **157** (2017).
31. M. Plummer, JAGS: A program for analysis of Bayesian graphical models using Gibbs sampling. *Proc. 3rd Int. Work. Distrib. Stat. Comput. (DSC 2003)*, 20–22 (2003).
32. R Development Core Team, R: A Language and Environment for Statistical Computing. *R Found. Stat. Comput. Vienna Austria* **0**, {ISBN} 3-900051-07-0 (2016).
33. Brown L.C. & Foster G. R, storm Erosivity Using Idealized Intensity Distributions. *Am. Soc. Agric. Biol. Eng.* **30**, 0379–0386 (1987).
34. C. J. Rosewell, Rainfall kinetic energy in eastern Australia. *J. Clim. Appl. Meteorol.* **25**, 1695–1701 (1986).
35. A. I. J. M. Van Dijk, L. A. Bruijnzeel, C. J. Rosewell, Rainfall intensity-kinetic energy relationships: A critical literature appraisal. *J. Hydrol.* **261**, 1–23 (2002).
36. K. Auerswald, M. Kainz, P. Fiener, Soil erosion potential of organic versus conventional farming evaluated by USLE modelling of cropping statistics for agricultural districts in Bavaria. *Soil Use Manag.* **19**, 305–311 (2003).
37. U.S. Department of Agriculture, 2012 National Resources Inventory. *2012 Natl. Resour. Invent.*, 5–8 (2015).
38. P. Panagos, *et al.*, The new assessment of soil loss by water erosion in Europe. *Environ. Sci. Policy* **54** (2015).
39. D. R. Montgomery, Soil erosion and agricultural sustainability. *Proc. Natl. Acad. Sci. U. S. A.* **104**, 13268–72 (2007).
40. O. Cerdan, *et al.*, Rates and spatial variations of soil erosion in Europe: A study based on erosion plot data. *Geomorphology* **122**, 167–177 (2010).
41. G. Grill, *et al.*, Mapping the world's free-flowing rivers. *Nature* **569**, 215–221 (2019).
42. J. A. Constantine, T. Dunne, J. Ahmed, C. Legleiter, E. D. Lazarus, Sediment supply as a

driver of river meandering and floodplain evolution in the Amazon Basin. *Nat. Geosci.* **7**, 899–903 (2014).

43. M. Meybeck, L. Laroche, H. H. Dürr, J. P. M. Syvitski, Global variability of daily total suspended solids and their fluxes in rivers. *Glob. Planet. Change* **39**, 65–93 (2003).
44. J. Milliman, K. Farnsworth, River Discharge to the Coastal Ocean: A Global Synthesis. *Cambridge Univ. Press. Cambridge, U.K*, 392pp (2011).
45. M. Vanmaercke, J. Poesen, J. Broeckx, J. Nyssen, Sediment yield in Africa. *Earth-Science Rev.* **136**, 350–368 (2014).
46. L. Oldeman, *The global extent of soil degradation*, ISRIC, Ed. (1994).
47. Z. G. Bai, D. L. Dent, L. Olsson, M. E. Schaepman, Proxy global assessment of land degradation. *Soil Use Manag.* **24**, 223–234 (2008).
48. M. Xiong, R. Sun, L. Chen, A global comparison of soil erosion associated with land use and climate type. *Geoderma* **343**, 31–39 (2019).
49. M. A. Nearing, Evaluating soil erosion models using measured plot data: Accounting for variability in the data. *Earth Surf. Process. Landforms* **25**, 1035–1043 (2000).
50. R. P. C. Morgan, M. A. Nearing, *Handbook of Erosion Modelling*, John Wiley & Sons, Ed. (2011) https:/doi.org/10.1002/9781444328455.
51. C. Alewell, P. Borrelli, K. Meusburger, P. Panagos, Using the USLE: Chances, challenges and limitations of soil erosion modelling. *Int. Soil Water Conserv. Res.* **7**, 203–225 (2019).
52. V. Naipal, C. Reick, J. Pongratz, K. Van Oost, Improving the global applicability of the RUSLE model - Adjustment of the topographical and rainfall erosivity factors. *Geosci. Model Dev.* (2015) https:/doi.org/10.5194/gmd-8-2893-2015.
53. F. Nachtergaele, *et al.*, "Global Land Degradation Information System (GLADIS) - An Information database for Land Degradation Assessment at Global Level" (2011).

SI Figure

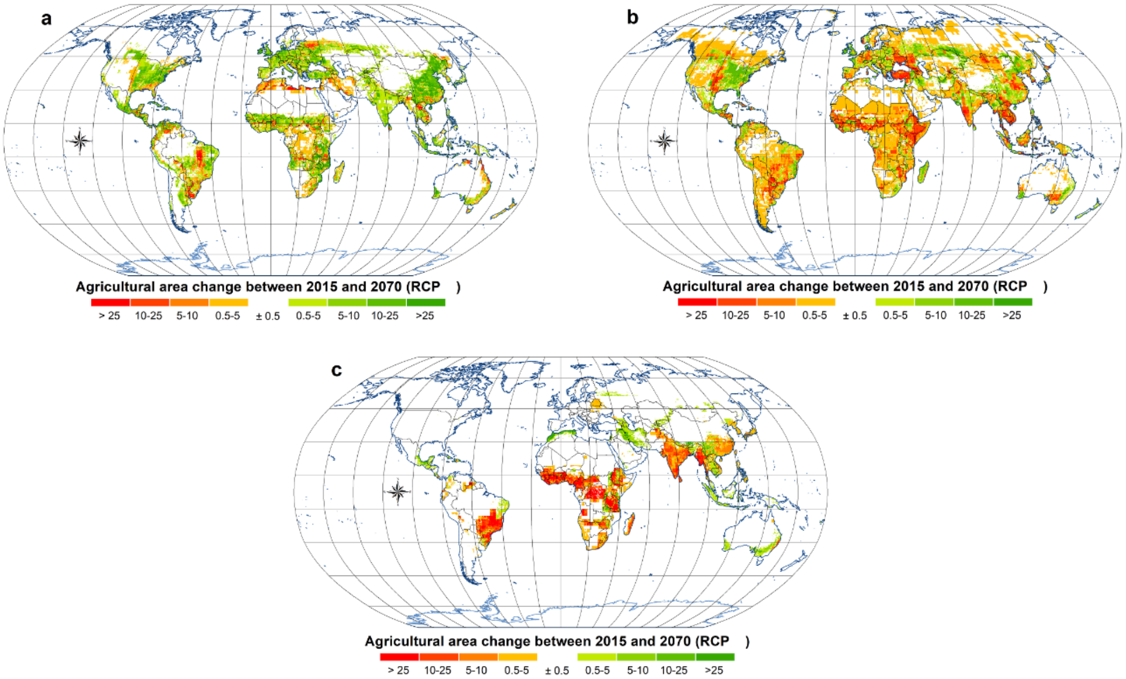


**Fig. S1.** Global annual changes in agricultural area between the reference period 2015 and the 2070 projections (SSP1-RCP2.6, IMAGE model; SSP2-RCP4.5, MESSAGE-GLOBIOM model; and SSP5-RCP8.5, REMIND-MAGPIE model).

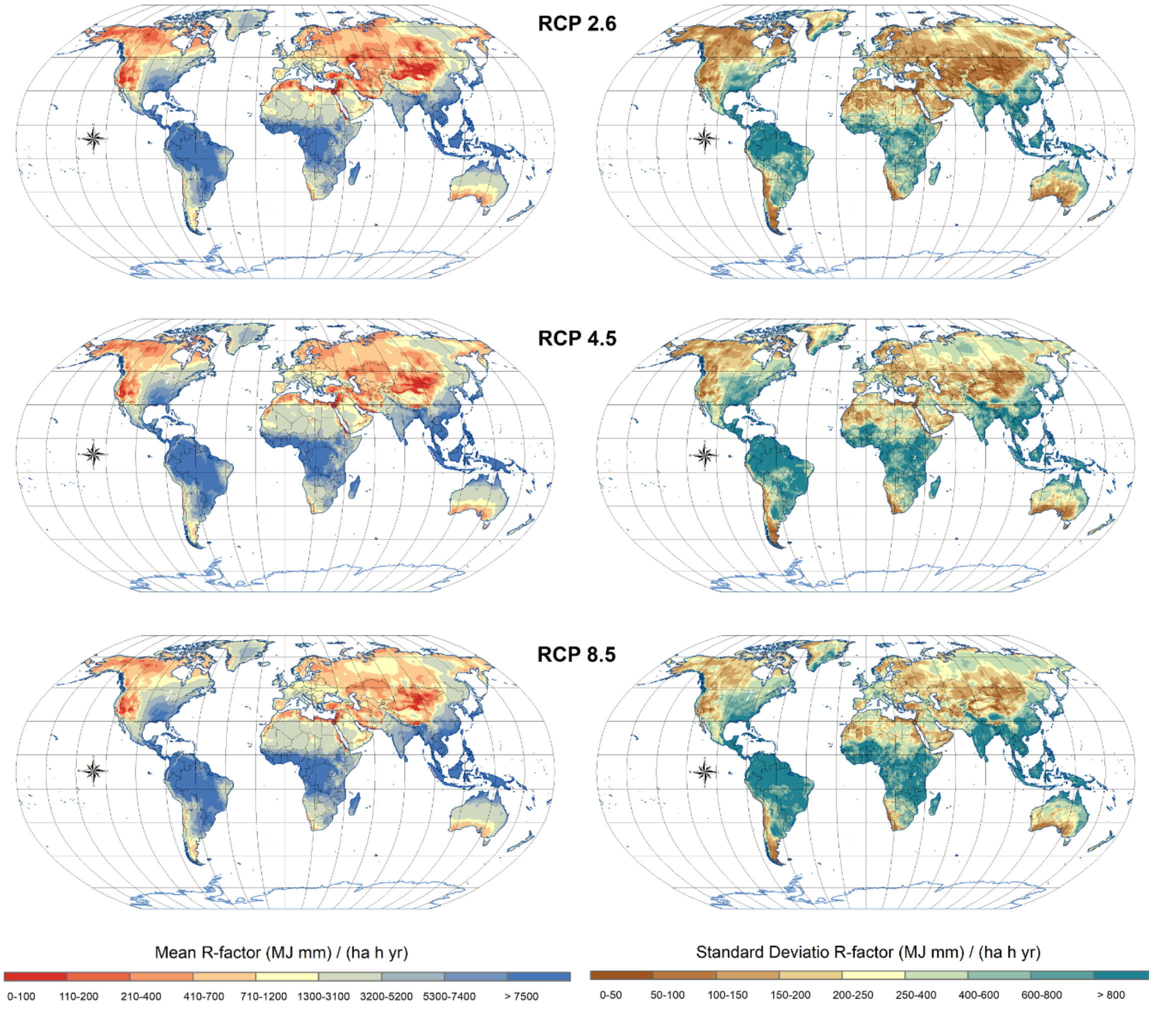


**Fig. S2.** Global rainfall erosivity map. The six panels illustrate the global patterns of the annual average rainfall erosivity R-factor (left panels) (spatial resolution 30 arc-seconds, ca. 1 x 1 km) and the standard deviation (right panels) obtained from the fourteen General Climate Models (GCMs).

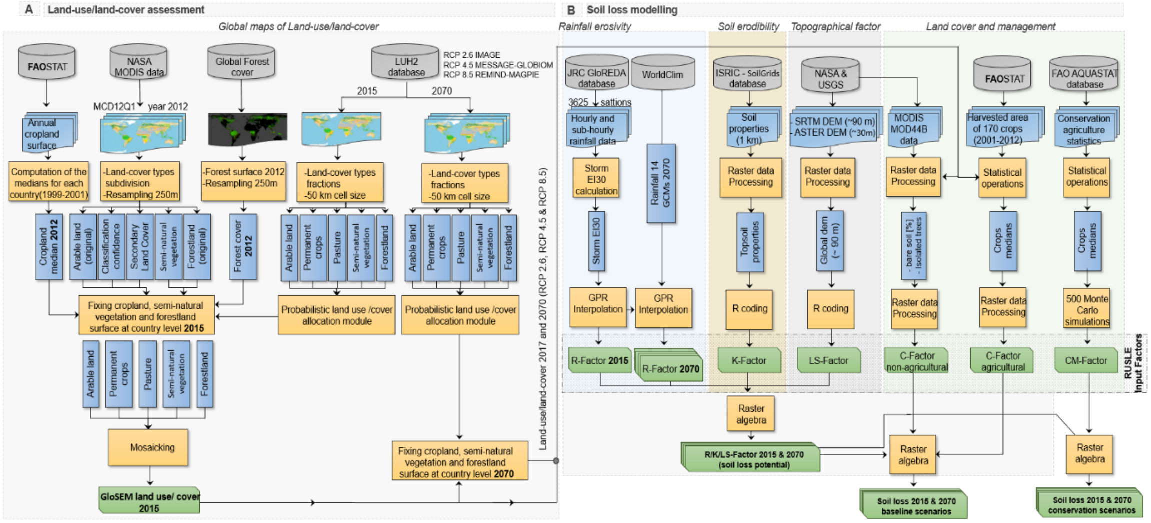


**Fig. S3.** Workflow of GloSEM platform updated to integrate future land use/ cover and climate changes. Schematic outline of the main processing steps carried out to a assess the extent, types and spatial distribution of the global croplands that are thoroughly defined combining satellite-derived land use and land cover information with agricultural inventory data, and b perform the high-resolution global soil erosion estimates.

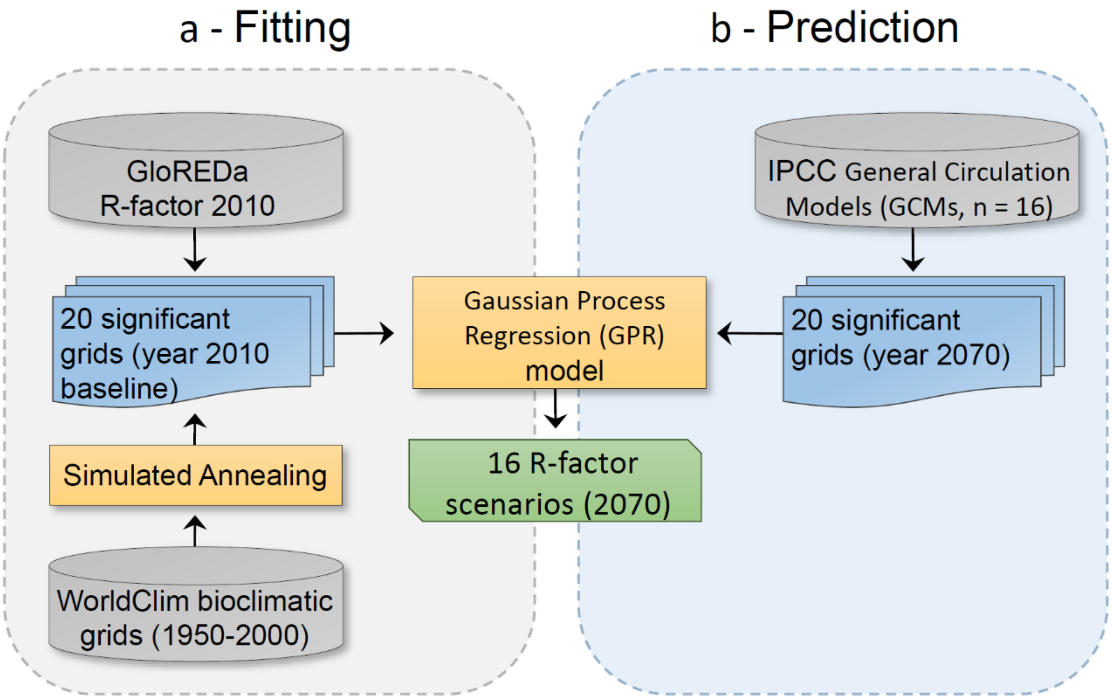


**Fig. S4.** Procedure followed to project future (2070) global rainfall erosivity. A panel: fitting part, B panel: prediction part. GloREDa refers to the Global Rainfall Erosivity Database (23).

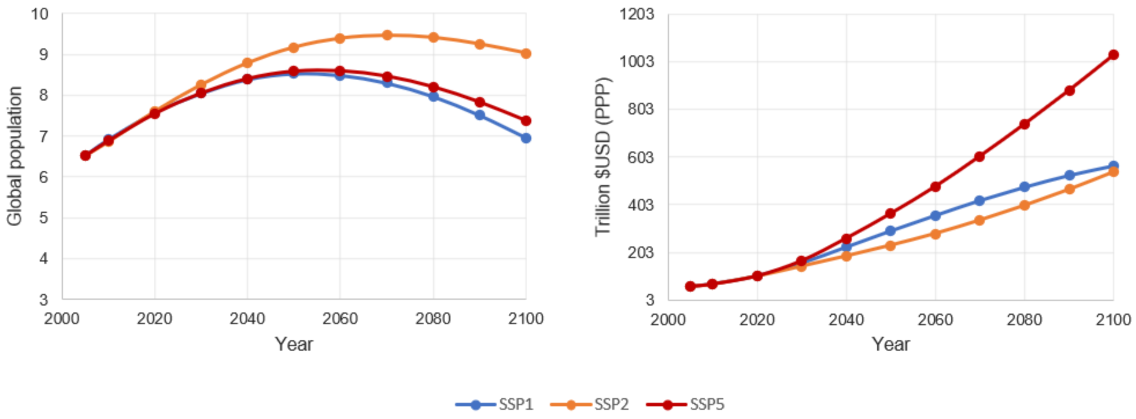


**Fig. S5.** Illustration of the three Shared Socioeconomic Pathways used in the present study. Left: global population in billions. Right: global gross domestic product in trillion US dollars on a purchasing power parity (PPP) (data source: Shared Socioeconomic Pathways database - Version 2.0).

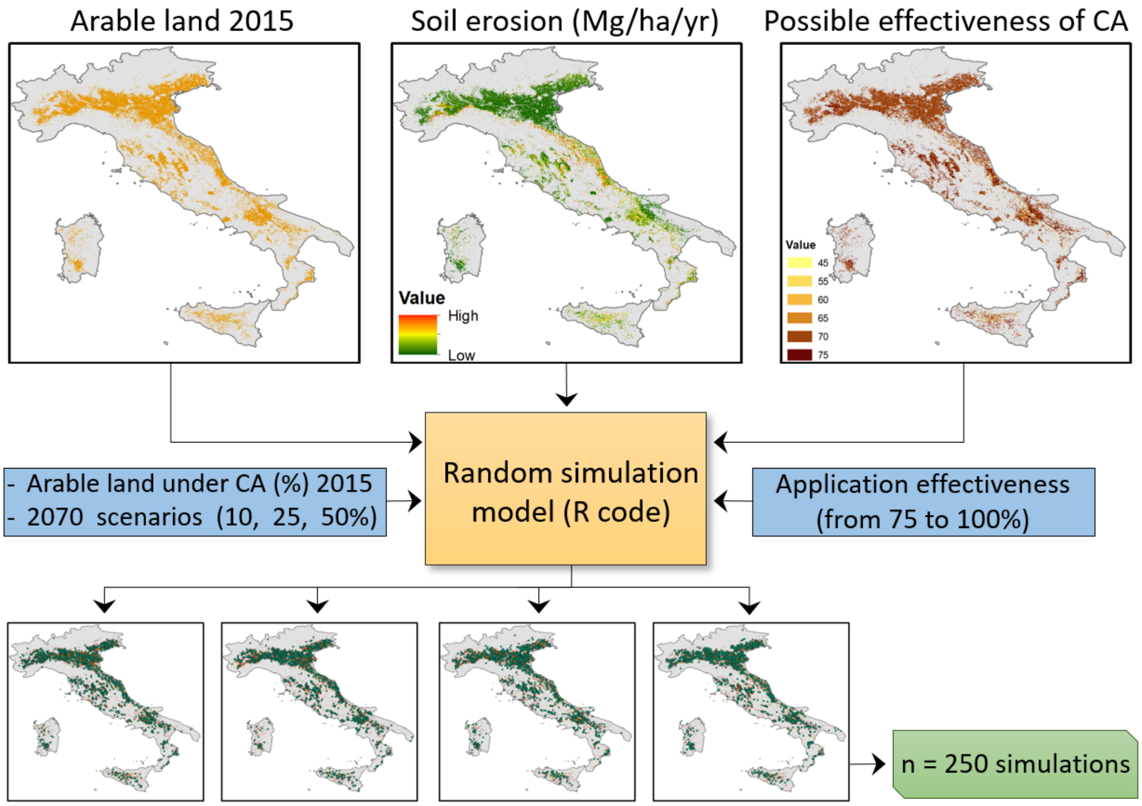


**Fig. S6.** Schematic outline of the main processing steps carried out to simulate 250 random conservation agriculture (CA) scenarios for each country.

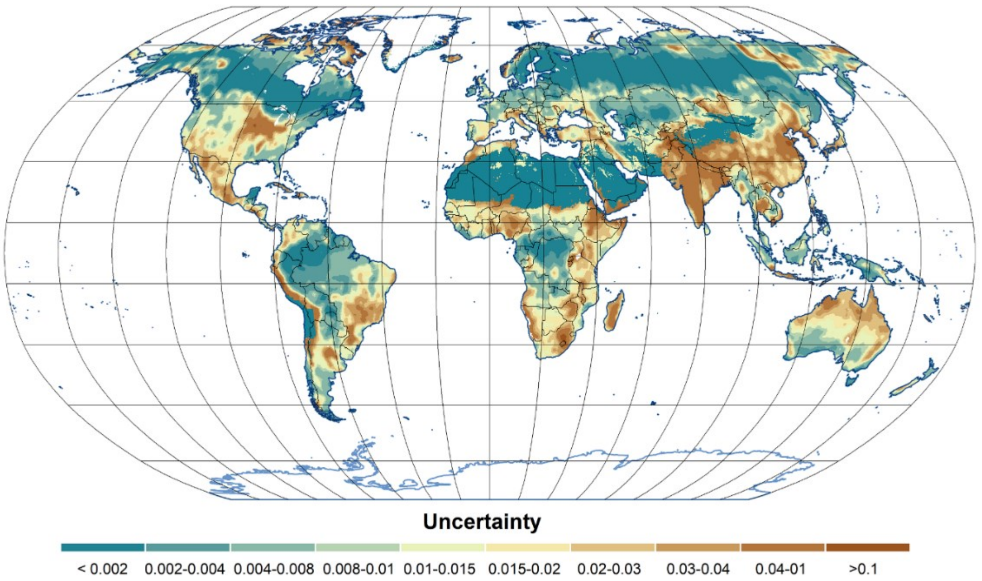


**Fig. S7.** The map of uncertainty presented as the standard deviation of the Markov Chain Monte Carlo (MCMC) simulated values.

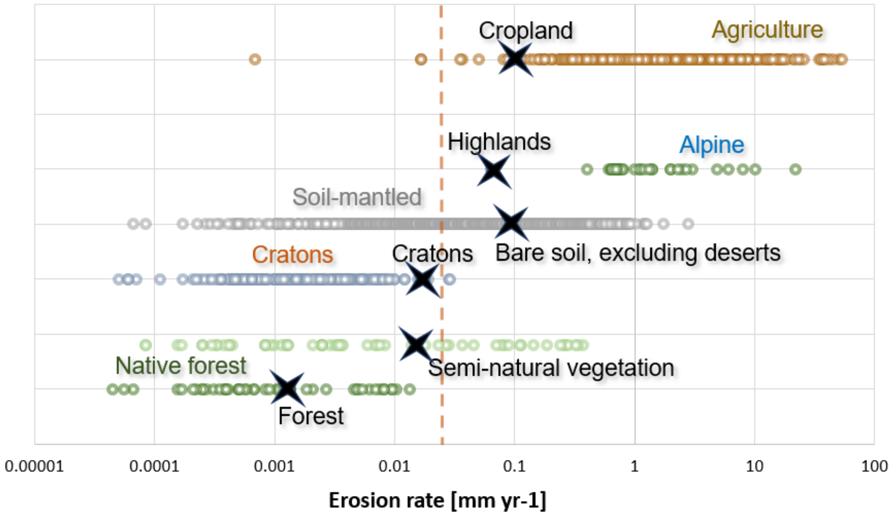


**Fig. S8**. Comparison of measured and modelled erosion rates. Representation of soil erosion rates measured on agricultural fields under conventional agriculture (n = 779), geologic erosion rates measured on alpine terrain (n = 44), soil-mantled landscapes (n = 1456), low gradient continental cratons (n = 218), grassland and scrublands (n = 63), native forests (n = 46) and averages of our predictions (indicated by an asterisk). Large parts of the measured data come from the study of Montgomery (39) integrated with data from other meta-analysis studies. The black 'starts' indicate the average values predicted by our model in 2015. The vertical red dotted line indicates average value of soil erosion.

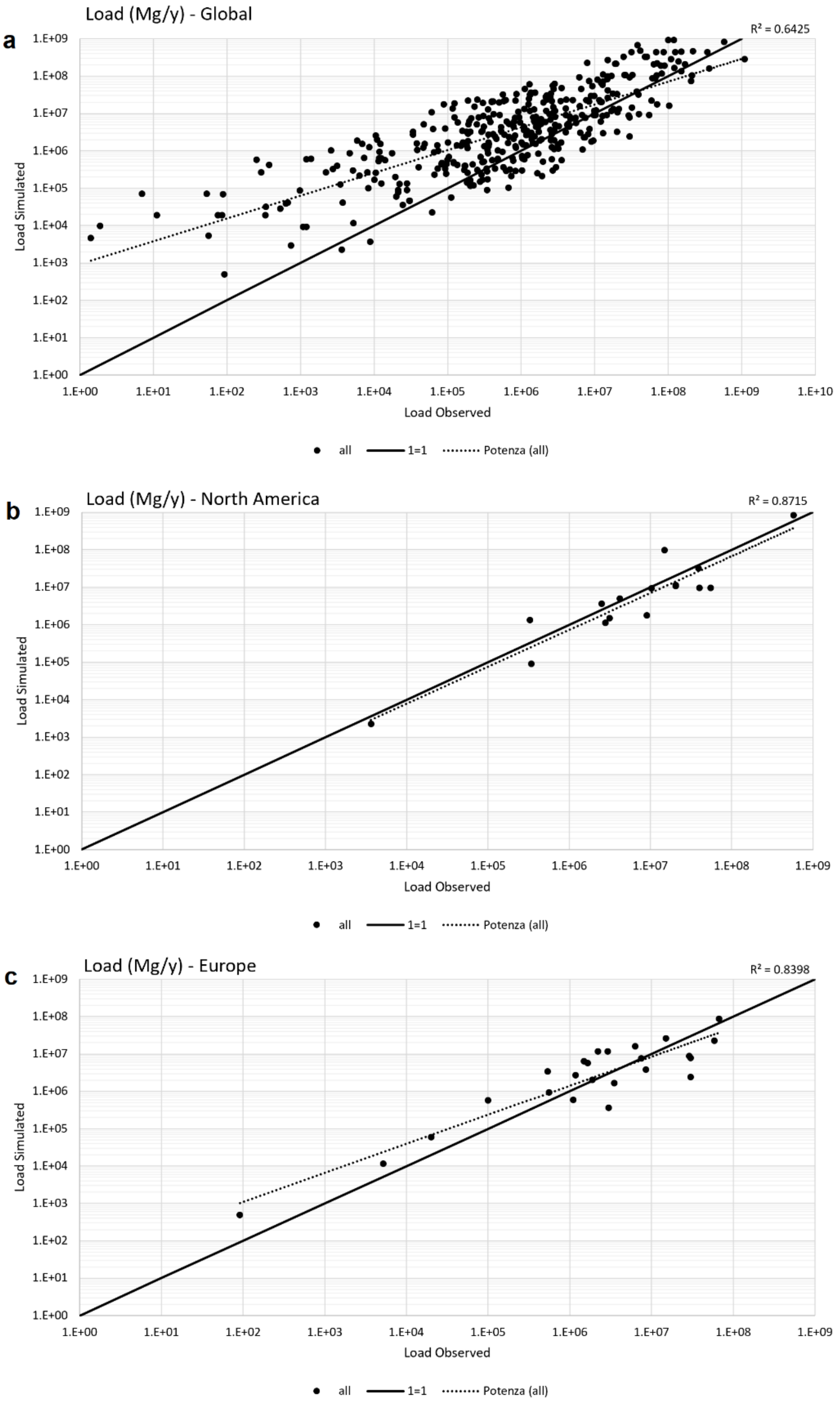


**Fig. S9**. Comparison of simulated load derived from GloSEM estimates against observed load (42–45) recorded at 398 gauging stations globally (panel a). The panels b and c report the same analysis considering the gauging stations in North America and Europe.

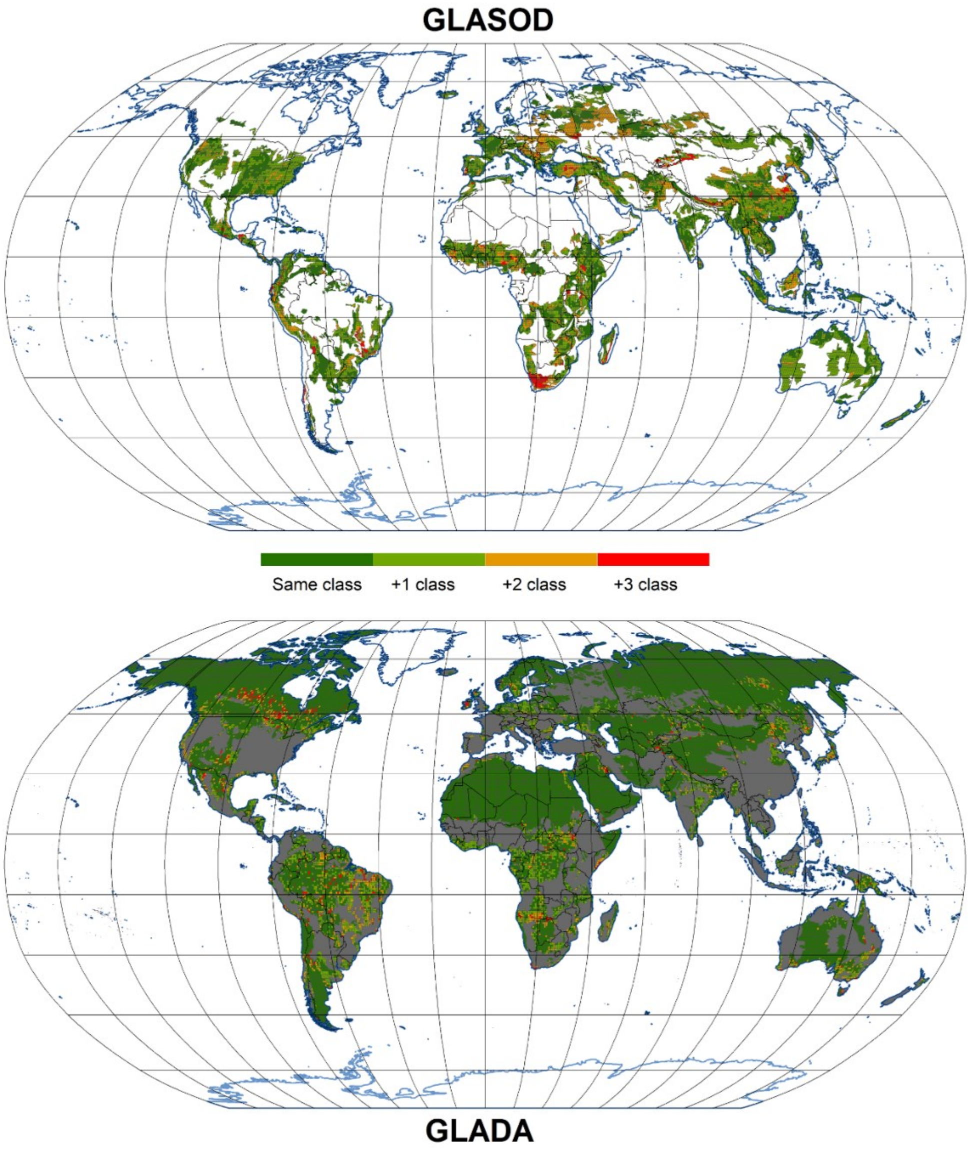


**Fig. S10**. Comparison of the spatial patterns of GLoSEM with the ones reported by previous global studies on soil erosion and land degradation made by the United Nations (UN). Panel a illustrates the Global Assessment of Soil Degradation (GLASOD). Panel b the Global Assessment of Land Degradation and Improvement (GLADA). Lands already reported by GLASOD as damaged by water erosion were masked out in GLADA. According to GLASOD original classification, the degree of damage of the areas affected by water erosion is reported in four classes ranging from low (W1) to severe (W4). GLoSEM and GLADA were ranked into four classes too using the quantile classification method.

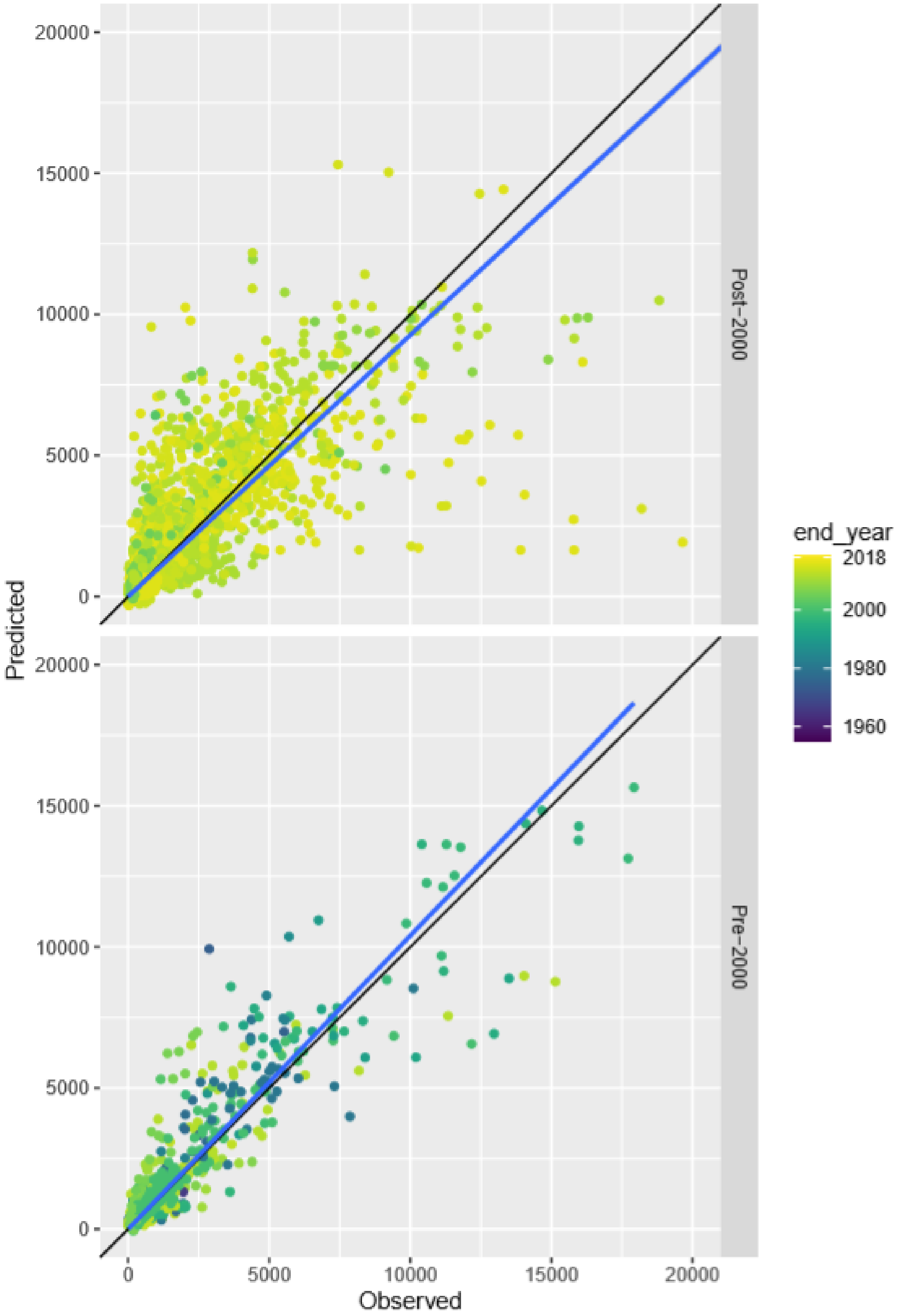


**Fig. S11**. Comparison of observed vs. predicted rainfall erosivity (R) values for the pre-2000 training set (lower graph) and for the post-2000 validation set (upper graph) (MJ mm$^{-1}$ ha$^{-1}$ yr$^{-1}$).

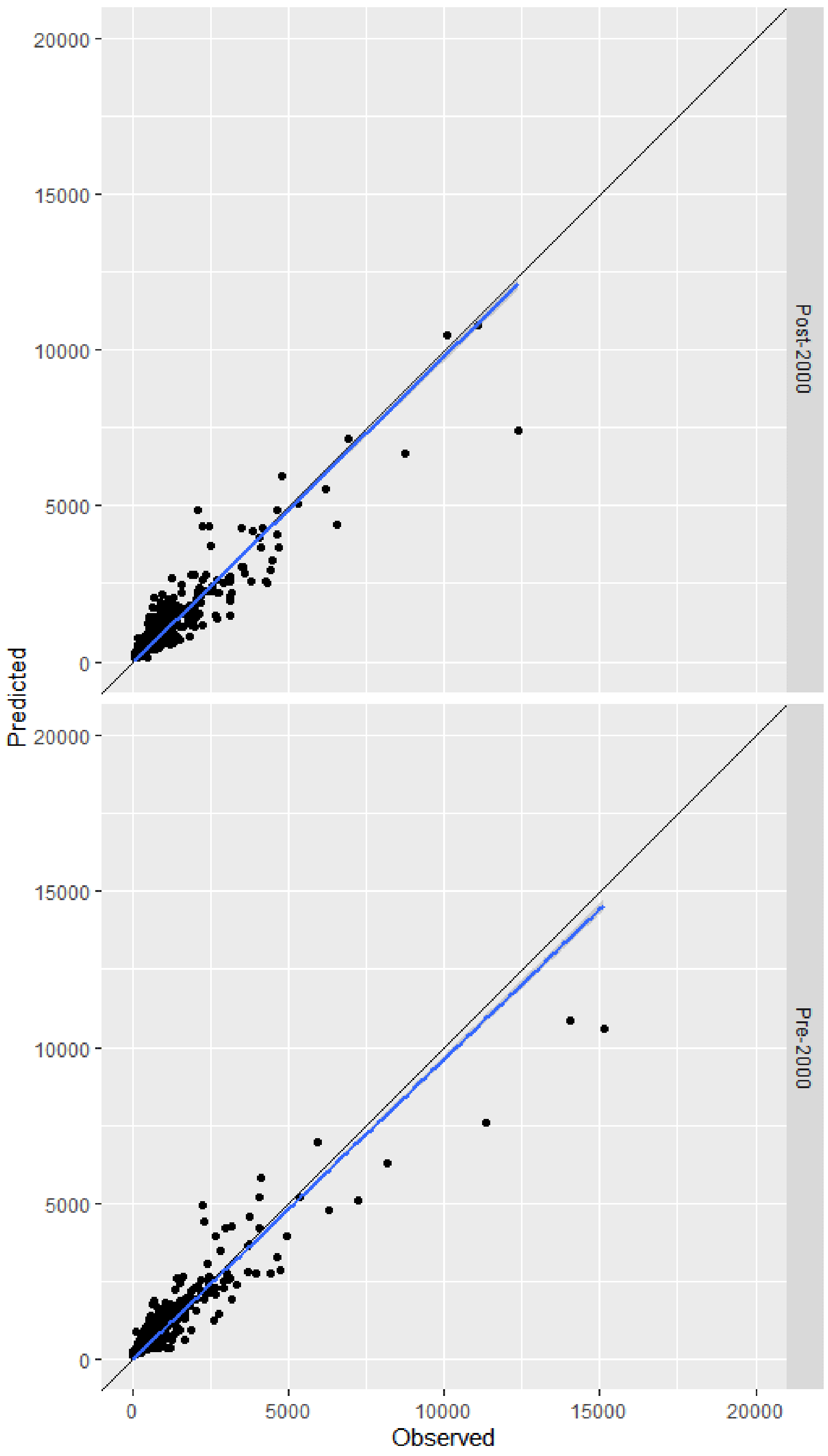


**Fig. S12**. Comparison of observed vs. predicted rainfall erosivity (R) values for the pre-2000 training set (lower graph) and for the post-2000 validation set (upper graph) (MJ mm$^{-1}$ ha$^{-1}$ yr$^{-1}$) using the stations for which the timeseries covers the whole observed period.

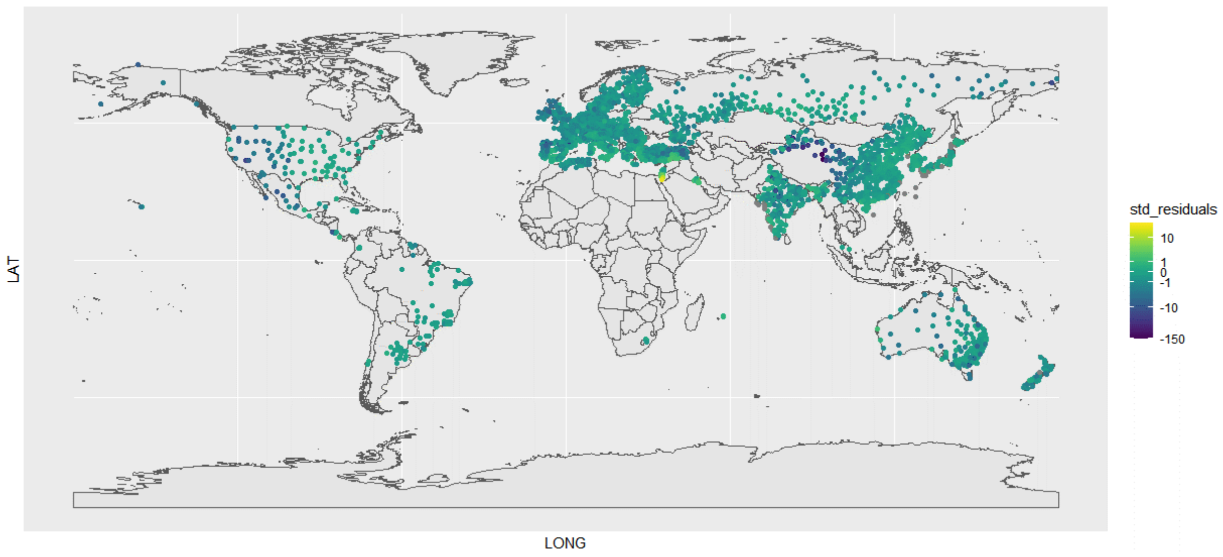


**Fig. S13**. Standard deviation of the residual proportion of the observed rainfall erosivity (R) values (MJ mm$^{-1}$ ha$^{-1}$ yr$^{-1}$).

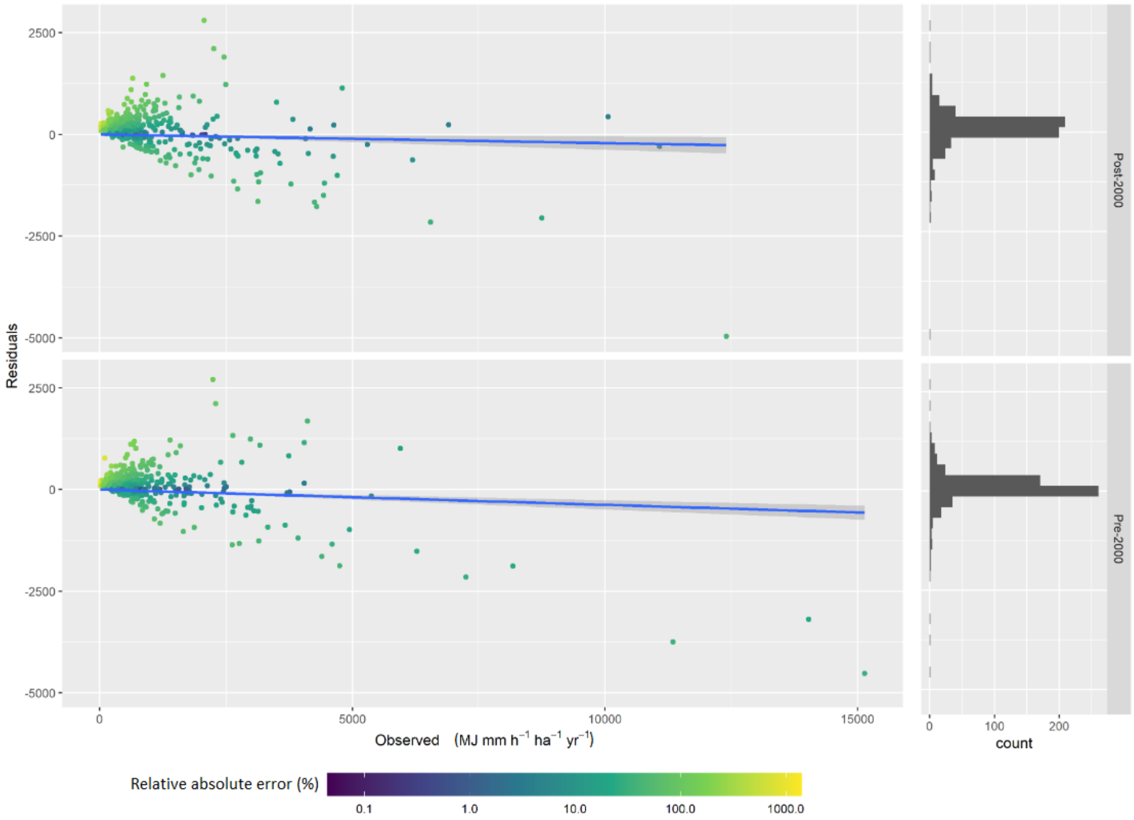


**Fig. S14**. Residuals plot for the Pre-2000 and the Post-2000 GPR predictions. The plots show the relation between the observed values and the difference between observed and predicted R-factor values (residuals). The blue trend line is a linear model fit. Points color represents the relative absolute error (expressed as a percentage) which is the ratio between the error and the observed value. On the right the histograms show the distribution of the residuals.

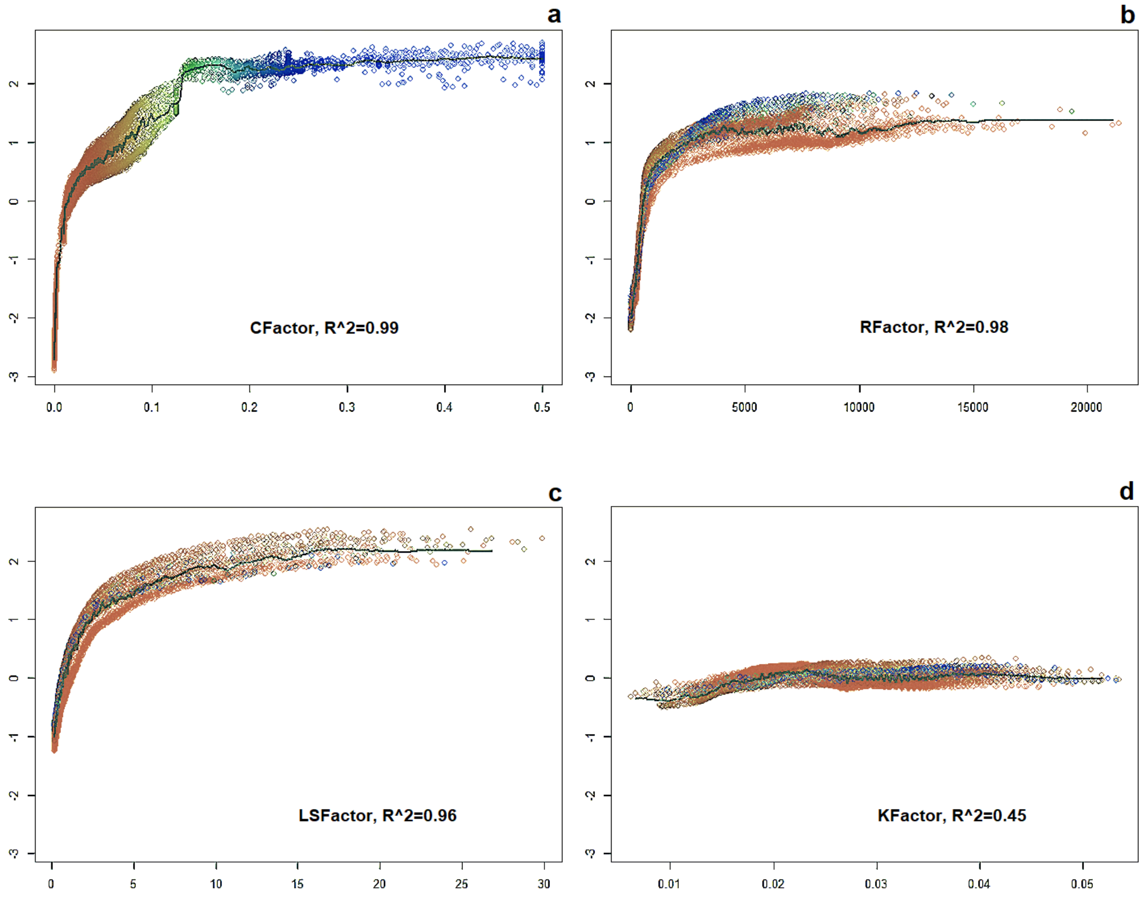


**Fig. S15.** Results of the sensitivity analyses (SA) performed to evaluate the influence of parameter estimation on uncertainty and the effect of parameters on model output. All four main model input parameters were considered: **(**a) the C-factor (cover and management); b the R-factor (rainfall-runoff erosivity; (c) the LS-factor (slope length and steepness factor); and (d) the K-factor (soil erodibility). The color of the four plots expresses the value of the most influential independent variable and allows to visualize the presence of interactions. The response is expressed in logarithmic scale on the vertical axis, the independent variable is plotted on the horizontal axis. More detail in can be found in Borrelli et al. (2).

SI Table

**Table S1**. Set of the 125 crop statistics used for the computation of the C-factor for croplands. The crops are grouped in thirteen crop groups according to their soil cover effectiveness. Crop data were provided by the Food and Agriculture Organization (FAO) (FAOSTAT database).

| **Crop Group 1: Cereal Grains** |
|---|
| Wheat |
| Barley |
| Sorghum |
| Millet |
| Oats |
| Rye |
| Triticale |
| Cereals, nes |
| Buckwheat |
| Grain, mixed |
| Fonio |
| Maize, green |
| Maize |
| Rice, paddy |
| **Crop Group 2: Legume Vegetables** |
| Soybeans |
| Beans, dry |
| Groundnuts, with shell |
| Chick peas |
| Cow peas, dry |
| Peas, dry |
| Pigeon peas |
| Pulses, nes |
| Lentils |
| Broad beans, horse beans, dry |
| Peas, green |
| Beans, green |
| Lupins |
| Vetches |
| Vegetables, leguminous nes |
| String beans |
| Bambara beans |
| **Crop Group 3: Root and Tuber Vegetables** |
| Cassava |
| Potatoes |
| Sugar beet |
| Yams |
| Sweet potatoes |
| Taro (cocoyam) |
| Roots and tubers, nes |
| Carrots and turnips |
| Vegetables and roots fodder |
| Ginger |
| Beets for fodder |
| Turnips for fodder |
| Swedes for fodder |

| **Crop Group 3: Root and Tuber** |
|---|
| Yautia (cocoyam) |
| Chicory roots |
| Carrots for fodder |
| **Crop Group 4: Fruiting Vegetables** |
| Tomatoes |
| Chillies and peppers, dry |
| Chillies and peppers, green |
| Eggplants (aubergines) |
| Okra |
| Pepper (piper spp.) |
| Artichokes |
| **Crop Group 5: Cucurbit Vegetables** |
| Watermelons |
| Cucumbers and gherkins |
| Pumpkins, squash and gourds |
| Melons, other (inc.cantaloupes) |
| Melonseed |
| **Crop Group 6: Bulb Vegetable** |
| Onions, dry |
| Garlic |
| Onions, shallots, green |
| Leeks, other alliaceous vegetables |
| Asparagus |
| **Crop Group 7: Leafy Vegetables** |
| Lettuce and chicory |
| Spinach |
| Anise, badian, fennel, coriander |
| Cauliflowers and broccoli |
| Cabbage for fodder |
| Cabbages and other brassicas |
| Quinoa |
| Vegetables, fresh nes |
| Tobacco, unmanufactured |
| ***Crop Group 8.* Forage, Fodder and Straw** |
| Mixed Grasses and Legumes |
| Forage and silage, maize |
| Forage and silage, legumes |
| Forage and silage, clover |
| Forage and silage, grasses nes |
| Forage products |
| Forage and silage, alfalfa |
| Forage and silage, rye grass |
| Forage and silage, green oilseeds |
| **Crop Group 9: Grapes and Hops** |
| Grapes |
| Hops |

*Continuation of* **Table S1**.

| **Crop Group 10: Oilseed Group** |
|---|
| Sunflower seed |
| Rapeseed |
| Sesame seed |
| Oilseeds nes |
| Linseed |
| Castor oil seed |
| Safflower seed |
| Mustard seed |
| Canary seed |
| Poppy seed |
| Jojoba seed |
| Seed cotton |
| **Crop Group 11: Fibre Crops** |
| Jute |
| Bastfibres, other |
| Manila fibre (abaca) |
| Flax fibre and tow |
| Agave fibres nes |
| Sisal |
| Fibre crops nes |
| **Crop Group 12: Berries Group** |
| Berries nes |
| Raspberries |
| Blueberries |
| Gooseberries |
| Cranberries |
| Currants |
| Strawberries |
| **Crop Group 13: Shrubs Herbs *and Spices Group*** |
| Coffee, green |
| Nutmeg, mace and cardamoms |
| Cinnamon (canella) |
| Vanilla |
| Maté |
| Cloves |
| Spices, nes |
| Sugar cane |
| Sugar crops, nes |
| Ramie |
| Tea (0.1-0.2) |
| Tea nes |
| Hemp tow waste |
| Hempseed |
| Peppermint |
| Pyrethrum, dried |
| |

| **Crop Group 14: Trees/Fruit Tree** |
|---|
| Almond |
| Almonds, with shell |
| Apples |
| Apricots |
| Areca nuts |
| Avocados |
| Bananas |
| Brazil nuts, with shell |
| Carobs |
| Cashew nuts, with shell |
| Cashewapple |
| Cherries |
| Cherries, sour |
| Chestnut |
| Cocoa, beans |
| Coconuts |
| Dates |
| Figs |
| Fruit, citrus nes |
| Fruit, fresh nes |
| Fruit, pome nes |
| Fruit, stone nes |
| Fruit, tropical fresh nes |
| Grapefruit (inc. pomelos) |
| Hazelnuts, with shell |
| Kapok fruit |
| Karite nuts (sheanuts) |
| Kiwi fruit |
| Kola nuts |
| Lemons and limes |
| Mangoes, mangosteens, guavas |
| Nuts, nes |
| Oil, palm fruit |
| Olives |
| Oranges |
| Papayas |
| Peaches and nectarines |
| Pears |
| Persimmons |
| Pineapples |
| Pistachios |
| Plantains |
| Plums and sloes |
| Quinces |
| Rubber, natural |
| Tallowtree seed |
| Walnuts, with shell |

**Table S2**. Crop groups and their corresponding base C-factors [dimensionless values] derived from literature.

| Crop Group | | | C-Factor |
|---|---|---|---|
| 1 | Cereal Grains | Various | 0.2 |
| | | Maize | 0.38 |
| | | Rice | 0.15 |
| 2 | Legume Vegetables | Various | 0.32 |
| 3 | Root and Tuber Vegetables | Various | 0.34 |
| 4 | Fruiting Vegetables | Various | 0.25 |
| 5 | Cucurbit Vegetables | Various | 0.25 |
| 6 | Bulb Vegetable | Various | 0.3 |
| 7 | Leafy Vegetables | Various | 0.25 |
| | | Tobacco | 0.5 |
| 8 | Forage, Fodder and Straw of Cereal Grains Group | Mixed-legumes | 0.15 |
| | | Mixed-grasses | 0.1 |
| 9 | Grapes and Hops | Grapes | 0.35 |
| | | Hops | 0.42 |
| 10 | Oilseed Group | Various | 0.25 |
| | | Cotton | 0.4 |
| 11 | Fibre Crops | Fibre Crops | 0.28 |
| 12 | Berries Group | Various | 0.15 |
| | | Strawberries | 0.2 |
| 13 | Shrubs Herbs and Spices Group | Shrubs Herbs and Spices | 0.15 |
| | | Coffee | 0.2 |

**Table S3**. Values of C-factor for the non-arable land. Values are based on land use/ land cover classified according to the MODIS MCD12Q1 Land Cover Type International Geosphere Biosphere Programme (IGBP). Class 21 was created after elaborations were made in this study.

| Class | IGBP Land-cover type | $C_{NA}$ |
|---|---|---|
| 0 | Water | No data |
| 1 | Urban and built-up | No data |
| 2 | Perennial crops (pixel explicit) | 0.01-0.385 |
| 3 | Managed pasture | 0.05-0.15 |
| 4 | Rangeland | 0.01-0.15 |
| 5 | Forest (primary and secondary) | 0.0001-0.003 |
| 6 | Semi-natural vegetation | 0.01-0.15 |
| 7 | Barren land/ rocks | No data |
| 8 | Snow and ice | No data |
| 9 | Permanent wetlands | No data |
| 10 | Riverine system | No data |

**Table S4**. Ranking of WorldClim variables according to the Simulated Annealing (SA) optimization. Variables are ranked according to their respective selection frequency.

| Parameter | Covariate explanation | Selection frequency | Included in the model (Y)es/(N)o |
|---|---|---|---|
| Prec_9 | Average precipitation (mm) in September | 100 | Y |
| Prec_3 | Average precipitation (mm) in March | 68 | Y |
| Tmin_3 | Average minimum temperature in March | 68 | Y |
| Bio_1 | Annual Mean Temperature | 63 | Y |
| Tmin_11 | Average minimum temperature in November | 63 | Y |
| Tmax_11 | Average maximum temperature in November | 60 | Y |
| Tmin_5 | Average minimum temperature in May | 58 | Y |
| Tmax_12 | Average maximum temperature in December | 56 | Y |
| Prec_10 | Average precipitation (mm) in October | 55 | Y |
| Tmax_4 | Average maximum temperature in April | 54 | Y |
| Tmin_2 | Average minimum temperature in February | 52 | Y |
| Tmax_1 | Average maximum temperature in January | 51 | Y |
| Tmin_12 | Average minimum temperature in December | 48 | Y |
| Tmax_10 | Average maximum temperature in October | 47 | Y |
| Tmin_9 | Average minimum temperature in September | 44 | Y |
| Prec_2 | Average precipitation (mm) in February | 30 | Y |
| Tmin_6 | Average minimum temperature in June | 21 | Y |
| Tmax_9 | Average maximum temperature in September | 13 | Y |
| Bio_7 | Temperature Annual Range (Max Temp. of Warmest Month - Min Temp. of Coldest Month) | 10 | Y |
| Tmin_8 | Average minimum temperature in August | 8 | Y |
| Bio_5 | Max Temperature of Warmest Month | 0 | N |

**Table S5**. List of the fourteen General Climate Models (GCMs) used for this study.

| Model | Link |
|---|---|
| BCC-CSM1-1 | http://forecast.bcccsm.ncc-cma.net/web/channel-43.htm |
| CCSM4 | http://www.cesm.ucar.edu/models/ccsm4.0/ |
| CNRM-CM5 | http://www.umr-cnrm.fr/spip.php?article126&lang=en |
| GFDL-CM3 | https://www.gfdl.noaa.gov/coupled-physical-model-cm3/ |
| GISS-E2-R | https://data.giss.nasa.gov/modelE/cmip5/ |
| HadGEM2-AO | https://portal.enes.org/data/enes-model-data/cmip5/resolution/resolution |
| HadGEM2-ES | https://portal.enes.org/data/enes-model-data/cmip5/resolution/resolution |
| IPSL-CM5A-LR | http://cmc.ipsl.fr/international-projects/cmip5/ |
| MIROC-ESM- | https://catalogue.ceda.ac.uk/uuid/f8271364bd914ad09803191057dfcaae |
| MIROC-ESM | http://adsabs.harvard.edu/abs/2011GMD.....4..845W |
| MIROC5 | https://www.isimip.org/gettingstarted/details/21/ |
| MPI-ESM-LR | https://www.mpimet.mpg.de/en/science/models/mpi-esm/ |
| MRI-CGCM3 | https://www.jstage.jst.go.jp/article/jmsj/90A/0/90A_2012-A02/_article |
| NorESM1-M | https://www.geosci-model-dev.net/6/687/2013/gmd-6-687-2013.html |

**Table S6**. Reduction of soil erosion due to the application of conservation agriculture (CA) used in this study compared to conventional agriculture/ tillage. The scheme follows the typical USLE effectiveness of contour farming in reducing soil erosion depending on the slope gradient.

| Slope (percent) | Reduction of soil erosion [%] |
|---|---|
| 0-2 | 70 |
| 2-5 | 75 |
| 5-8 | 75 |
| 8-12 | 70 |
| 12-16 | 65 |
| 16-20 | 60 |
| 20-25 | 55 |
| >25 | 45 |

**Table S7**. Descriptive statistics of the soil erosion rates reported by Xiong's et al. (48) and GloSEM aggregated by land use/ cover types.

| | Measured | | | Modeled | | |
|---|---|---|---|---|---|---|
| | Average | Median | SD | Average | Median | SD |
| | [Mg $ha^{-1}$ $yr^{-1}$] | | | | | |
| Cropland | 22.1 | 4.9 | 47.0 | 25.6 | 1.8 | 84.9 |
| Fallow land | 103.8 | 30.7 | 181.6 | 96.6 | 45.7 | 128.1 |
| Forestland | 0.4 | 0.1 | 0.7 | 0.9 | 0.3 | 1.2 |
| Semi-natural vegetation | 0.8 | 0.1 | 2.2 | 1.8 | 0.4 | 3.6 |

**Table** S8. Ordinary least squares coefficients (B) of global models for the assessment of rainfall erosivity (R-factor).

| | Naipal et al. (52) | | | Nachtergaele et al. (53) | | |
|---|---|---|---|---|---|---|
| | B | std. Error | p-value | B | std. Error | p-value |
| Intercept | 1458.2 | 279.3 | <0.001 | −1149.0 | 141 | <0.001 |
| Regression coefficient | 0.15 | 0.12 | 0.187 | 0.73 | 0.04 | <0.001 |
| Observations | 3530 | | | 3530 | | |
| R2 | 0.155 | | | 0.385 | | |

| | Yang et al. (18) | | | GloSEM (22) | | |
|---|---|---|---|---|---|---|
| | B | std. Error | p-value | B | std. Error | p-value |
| Intercept | 1223.8 | 225.7 | <0.001 | −204.2 | 81.2 | 0.012 |
| Regression coefficient | 0.2 | 0.08 | 0.009 | 1.13 | 0.05 | <0.001 |
| Observations | 3530 | | | 3530 | | |
| R2 | 0.207 | | | 0.811 | | |
